# Multi‐omics insights for deciphering prognosis‐related T cell subsets in hepatocellular carcinoma

**DOI:** 10.1002/ctm2.70708

**Published:** 2026-06-01

**Authors:** Guangzu Cui, Erya Hu, Qingping Peng, Xin Zhou, Yu Zhao, Haicong Liu, Xinwen Wang, Yihong Chen, Hong Shen, Shan Zeng, Jiayao Ma

**Affiliations:** ^1^ Department of Oncology Xiangya Hospital Central South University Changsha Hunan China; ^2^ National Clinical Research Center for Geriatric Disease Xiangya Hospital Central South University Changsha Hunan China

**Keywords:** hepatocellular carcinoma, multi‐omics, prognosis, T cells, tumour immune microenvironment

## Abstract

**Background:**

Hepatocellular carcinoma (HCC) is one of the leading causes of tumour‐related death. T cells and cytokines play a critical role in tumour progression, but the T cell landscape correlated with HCC prognosis remains undepicted.

**Methods:**

The prognostic significance of intra‐tumoural immune cells, chemokines and cytokines were analysed using mass cytometry, bulk RNA sequencing and scRNA‐seq data with survival information. The signature of CD4^+^CD8^+^ double positive T (DPT) cells was constructed using scRNA‐seq and quantified by ssGSEA scores, whose association with the response to atezolizumab plus bevacizumab was evaluated. Cellular cross‐talk and spatial patterns were analysed by scRNA‐seq and spatial transcriptomics. Flow cytometry, gene knockdown, transwell migration, co‐culture assays, qPCR and wound healing assay were performed to further validate the DPT‐associated niche.

**Findings:**

Higher intra‐tumoural levels of DPT cells, CD45RA^+^CD4^+^ conventional T cells, HBEGF and CX3CR1 were associated with unfavourable prognosis in HCC. In contrast, higher infiltration of CD161^+^CD45RA^−^CD4^+^ conventional T cells and CD8^+^ T cells correlated with prolonged survival. CD45^+^EpCAM^+^ and CD45^+^α‐SMA^+^ cells were more frequent in short‐term survivors. DPT infiltration was identified across HCC multi‐cohorts and syngeneic mouse models. In patients receiving atezolizumab plus bevacizumab, responders exhibited higher DPT ssGSEA scores than non‐responders. Multi‐omics analyses indicated cross‐talk and spatial association of DPT cells with capillary‐associated endothelial cells, supporting a pro‐tumour niche. HBEGF was positively correlated with DPT cells and highly expressed in endothelial compartments. Endothelial‐derived HBEGF knockdown reduced DPT migration. Moreover, DPT co‐culture increased expression of signatures associated with immunosuppressive checkpoints, chemokine signalling, epithelial–mesenchymal transition and stemness in Hep3B cells and promoted their migration.

**Conclusion:**

Our findings depicted the prognostic immune landscape of HCC by identifying distinct T cell populations and molecular interactions. DPT cells emerged as a critical biomarker for poor prognosis, and the endothelial‐derived HBEGF–DPT axis could represent a potential therapeutic target.

## Graphical abstract

- An integrated multi‐omics analysis delineates the prognostic landscape of T‐cell subsets in hepatocellular carcinoma.
- DPT cells and HBEGF‐related signals are associated with poor survival in hepatocellular carcinoma.
- Endothelial‐derived HBEGF recruits DPT cells, which in turn up‐regulate the EMT signatures and drive the migration of hepatocellular carcinoma cells.
- DPT‐related signatures are associated with response to atezolizumab plus bevacizumab therapy.

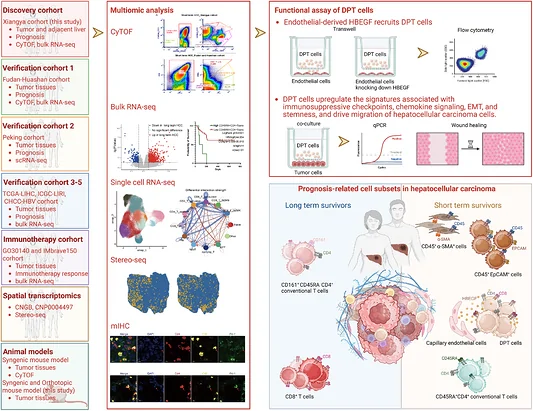

## 1 INTRODUCTION

Hepatocellular carcinoma (HCC) is the predominant histological type of primary liver cancer.^1^ For patients with early‐stage HCC, surgical resection remains the preferred treatment, but postoperative recurrence remains common, with a 5‐year recurrence rate of 50–70%.^2^ For patients with intermediate‐stage HCC, transarterial chemoembolisation is the standard of care.^2^ For patients with advanced‐stage HCC, tyrosine kinase inhibitors and immune checkpoint inhibitors have improved clinical outcomes.^2^ However, the number of patients who benefit is limited, and the 5‐year overall survival rates for liver cancer is below 20%.^3^ Recent studies have also shown that lactylation‐ and metabolism‐related mechanisms contribute to HCC progression and prognosis.^4^, ^5^, ^6^

The tumour microenvironment (TME) is composed of a variety of immune cell populations. Some immune cell clusters can clear tumour cells, while some immune cell clusters will promote tumour progression and affect the therapeutic efficacy.^7^, ^8^ Therefore, identifying key cell subsets and shifting them from a tumour‐promoting to a tumour‐suppressing state, or directly targeting key tumour‐promoting cell subsets, will provide strategies for tumour treatment based on the immune microenvironment. Single‐cell technologies, including single‐cell RNA sequencing (scRNA‐seq) and cytometry by time‐of‐flight (CyTOF), enable high‐resolution characterisation of cellular heterogeneity within the TME and facilitate the discovery of novel cell subsets relevant to precision immunotherapy and clinical translation. ScRNA‐seq provides single‐cell transcriptomic data, while CyTOF enables ultra‐high‐resolution detection of single‐cell proteomics, allowing for further understanding of the diversity of immune cells at the protein level. Recent studies have emphasised the heterogeneity of immune cells among cancerous tissues, cancerous border tissues and adjacent tissues in relation to the prognosis through CyTOF.^9^, ^10^, ^11^ In addition, the immune landscape of HCC due to different aetiologies has also been described.^12^ IFNγ Tc17 subset has been demonstrated to have pro‐tumoural characteristics.^13^ However, no study has been conducted to compare differences in the immune microenvironment based on patient survival through CyTOF.

In this study, we performed high‐dimensional CyTOF analysis on tumour samples from HCC patients with contrasting clinical outcomes, long‐term survivors and short‐term survivors, to identify survival‐associated cell subsets within the TME. By examining the distribution of immune cells and comparing their proportions between tumour and tumour‐adjacent tissues, we aimed to identify immune cell subsets with significant differences and characterise their functions. Further, single‐cell sequencing, transcriptomics sequencing and multiplex immunofluorescence staining were performed to validate the role of cell subsets of interest. Additionally, we employed FlowSOM automatic clustering to identify novel cell subsets, which may contribute to differential survival outcomes and serve as potential biomarkers or therapeutic targets. Altogether, these findings advance our understanding of the cellular complexity underlying HCC progression and highlight the value of single‐cell approaches in identifying prognostically relevant cell subsets.

## 2 METHODS

### 2.1 Patient samples of the Xiangya cohort and cell isolation

Fresh tumour tissues (*n* = 16) along with 7 paired adjacent non‐tumour tissue samples were collected for CyTOF analysis and bulk RNA‐seq from 16 patients who underwent surgical resection for HCC at Xiangya Hospital. Moreover, an independent cohort of HCC patients with resected and archival formalin‐fixed paraffin‐embedded (FFPE) tissue samples was also collected. Only cases with a confirmed diagnosis of HCC and available prognostic information were included. This study was approved by Ethics Committees of Xiangya Hospital. Leukocytes were isolated from tissues by tissue digestion and density gradient centrifugation for CyTOF, as previously described.^11^ All samples were anonymously coded. Clinical characteristics of Xiangya cohorts are presented in Table S1.

### 2.2 Publicly available data collection

CyTOF data and transcriptome data of human specimens were obtained from Huashan Hospital (*n* = 35), and mass spectrometry data of mice were obtained from the Department of Hepatobiliary Surgery, Dongfang Hospital. Clinical characteristics of Fudan–Huashan cohort are presented in Table S2. scRNA‐seq data of HCC patients were obtained from Zhang Zemin's team, deposited in the National Genomics Data Center (Beijing, China) (https://ngdc.cncb.ac.cn/) under BioProject ID PRJCA007744, and subsequently analysed (*n* = 60).^14^ In addition, nine patients with paired scRNA‐seq profiles from blood, tumour and adjacent non‐tumour tissues were included for additional analyses. The clinical information and groupings of the enrolled samples are provided in the Table S3.

We retrieved data of 368 patients in the TCGA‐LIHC cohort from the TCGA website (https://portal.gdc.cancer.gov/repository). Data of 159 Chinese HBV‐associated HCC cases in the CHCC‐HBV cohort were obtained from the NODE database (https://www.biosino.org/node). In addition, data from 232 patients in the LIRI‐JP liver cancer project were downloaded via the ICGC portal (https://dcc.icgc.org/projects/LIRI‐JP). Only samples with a confirmed diagnosis of HCC and available prognostic information were included.

Clinical data and pretreatment tumour RNA‐seq data for patients treated with atezolizumab plus bevacizumab were obtained from the biomarker datasets of the GO30140 and IMbrave150 trials in the European Genome‐phenome Archive (https://ega‐archive.org/; EGAS00001005503).^15^ GO30140 was an open‐label, multi‐centre phase 1b study that enrolled patients with unresectable HCC who had not received previous systemic therapy. The HCC cohorts included a single‐arm atezolizumab plus bevacizumab cohort (group A) and a randomised cohort comparing atezolizumab plus bevacizumab with atezolizumab alone (group F). IMbrave150 was a global, open‐label, randomised phase 3 trial in patients with previously untreated unresectable HCC, an ECOG performance status of 0 or 1, Child‐Pugh class A liver function, and at least one measurable untreated lesion according to RECIST v1.1. In the present study, only pretreatment samples with available transcriptomic data were included. After merging the GO30140 and IMbrave150 cohorts, batch effects between trials were corrected using the ComBat function in the R package sva (v3.46.0).^16^ Treatment response was dichotomised according to RECIST v1.1: complete or partial response was grouped as objective response, whereas stable or progressive disease was grouped as non‐response. Samples with non‐evaluable response or missing response information were excluded. A total of 247 patients were included in the final analysis, including 81 responders and 166 non‐responders.

Stereo‐seq spatial transcriptomic data from two HCC patients (HCC01 and HCC03) reported by Li et al. were included for analysis and are available in the China National GeneBank Sequence Archive (https://ngdc.cncb.ac.cn/gsa/) under accession code CNP0004497.^17^

### 2.3 CyTOF staining and data analysis

CyTOF analyses in the Xiangya and Fudan–Huashan cohorts were performed using cohort‐specific antibody panels, which are provided in Tables S4 and S5, respectively. Antibodies conjugated to distinct metal isotopes were used to profile immune cell subsets in tumour samples. After normalisation by the service provider (StarionX1; Polaris Biology), CyTOF datasets were generated as FCS 3.0 files and then subjected to manual gating with FlowJo v10 software (BD Biosciences). For quality control, EQ beads were first removed (Cleanup_Beads), followed by exclusion of events with abnormal event length (Cleanup_Event_Length) and events with atypical residual parameters (Cleanup_Residual_Center and Cleanup_Residual_Offset). Nucleated live cells were then retained for downstream analyses, and only samples with sufficient high‐quality events after cleanup gating were included in subsequent analyses. Immune cell populations were subsequently identified by sequential gating of CD45^+^ leukocytes and their major sublineages, as illustrated in Figure S1B–D. In the T‐cell compartment, CD4^+^CD8^+^ double positive T (DPT) cells were defined as CD45^+^CD3^+^CD4^+^CD8^+^ cells. FlowSOM clustering was performed using the R/Bioconductor package FlowSOM (v2.12.0), and data were visualised using t‐distributed stochastic neighbour embedding (t‐SNE) with Maxpar Pathsetter (De Novo Software). Given the differences in CyTOF panels and analytical workflows, the Xiangya and Fudan–Huashan cohorts were processed and analysed separately.

### 2.4 Tissue bulk RNA‐sequencing and analysis

Transcriptome sequencing was conducted by BGI‐Shenzhen. For the Xiangya and Fudan–Huashan cohorts, both raw count data and FPKM expression matrices were available. The correspondence between transcriptome samples and CyTOF samples is provided in Tables S6 and S7. Differential expression analysis was carried out in R (v4.2.3) with DESeq2 (v1.38.3) using raw count data. Genes with an adjusted *p* value < .05 and an absolute log2 fold change > 1 were defined as differentially expressed.^18^

Univariate and multivariable Cox proportional hazards regression analyses were conducted with the survival package (v3.5‐5). For univariate Cox analysis, scaled FPKM values were entered as continuous variables. Multivariable models were adjusted for sex, age, postoperative treatment and HBV status. Kaplan–Meier survival curves were generated with survminer (v0.4.9). For cytokine‐related Kaplan–Meier analyses, optimal cutoffs were determined using survminer (v0.4.9). Correlations between mass cytometry‐derived features and gene expression were evaluated by Spearman analysis using ggcor (v0.9.8.1).

### 2.5 Construction of orthotopic and de novo HCC mouse models

All procedures involving animals were reviewed and authorised by the Ethics Committee of Xiangya Hospital, Central South University.

For the orthotopic HCC model, five 7‐week‐old male BALB/c mice were purchased from Hunan SLAC Laboratory Animal Co. Under general anaesthesia and aseptic conditions, the liver was exposed through a midline abdominal incision, and 5 × 10^5^ H22 cells were injected under the capsule of the left hepatic lobe. The abdominal wall was then closed in layers. Mice were observed after surgery and sacrificed 21 days later, and tumour tissues were collected for subsequent analyses.

For the spontaneous HCC model, male and female C57BL/6 mice were purchased from Hunan SLAC Laboratory Animal Co and bred in the breeding facility of the Department of Laboratory Animal Science, Xiangya Hospital. Fourteen‐day‐old offspring were used for model induction. Five pups received a single intraperitoneal injection of diethylnitrosamine (25 mg/kg). Starting at 28 days of age, carbon tetrachloride (CCl_4_, 5 mg/kg) was administered intraperitoneally twice per week. Mice were sacrificed at 6 months, and liver tumours were collected for subsequent analyses. During the experiment, animals were monitored regularly and were euthanised earlier if they developed marked abdominal distension, severe ascites, impaired mobility, obvious weight loss or other signs of distress.

### 2.6 Multiplexed immunochemistry

FFPE tissue sections from the Department of Pathology of Xiangya Hospital were heated at 65°C for 20 min. The tissue sections were then subjected to deparaffinisation and rehydration by being sequentially immersed in Clearing Agent (Xylene) for 5 min three times, 100% ethanol for 3 min twice, 95% ethanol for 3 min twice, 80% ethanol for 3 min twice and distilled water for 3 min twice. 1X Tris–EDTA (10 mM Tris Base, 1 mM EDTA, pH 9.0) was used to retrieve antigens in a microwave oven, maintained at a sub‐boiling temperature for 15 min. Endogenous peroxidase was blocked by 3% H_2_O_2_ for 10 min at room temperature (RT). Before primary antibody incubation, tissue sections were blocked with 5% BSA (Biosharp) for 10 min at RT. The tissue sections were incubated with anti‐CD8 alpha (ab237709; Abcam), anti‐CD3(ab135372; Abcam), anti‐CD45 (ET7111‐03; HUABIO), anti‐CD4 (ab133616; Abcam), anti‐PD‐1 (ab309363; Abcam), anti‐α‐SMA (awa10574; Abiowell), anti‐EpCAM (ab223582; Abcam), anti‐Cd4 (ab183685; Abcam) and anti‐Cd8 alpha (ab217344; Abcam), respectively, at 4°C, overnight in a staining tray. The next morning, tissue sections were washed in 1× TBST and incubated with a secondary antibody (Abiowell), in terms of the species of the primary antibody for 30 min at RT. Followed by a quick wash in 1× TBST was followed by incubation with Opal690 (1:200), Opal620 (1:400), Opal570 (1:400) and Opal520 (1:400), respectively, for 30 min at RT. The tissue sections were again antigenically unmasked and blocked, and the next staining round was performed. Sections were mounted with coverslips using ProLong® Gold Antifade Reagent with DAPI. Sections were scanned by PerkinElmer Vectra Polaris Automated Quantitative Pathology Imaging System for spectral separation using inForm software.

### 2.7 scRNA‐seq analysis

scRNA‐seq data were mainly analysed using Seurat (v5.3.0).^19^ Cell quality was evaluated using three criteria: cells were retained only if the total UMI count per cell was <10 000, the number of detected genes ranged from 500 to 6000, and the proportion of mitochondrial gene expression was <5%. Doublets were identified and removed using scDblFinder (v1.20.2), and predicted doublet clusters were further inspected based on aberrant co‐expression of marker genes from distinct major cell types.^20^ Batch correction and data integration were performed with Harmony (v1.2.3) using origin.idents as the batch variable.^21^ PCA was conducted on the integrated assay, and the number of PCs used for downstream analyses was determined empirically based on the elbow plot (20–30 PCs, corresponding to the inflection point where the curve began to plateau). Using the selected principal components, a shared nearest neighbour graph was built with Seurat FindNeighbors, followed by community detection via FindClusters at an empirically determined resolution to define major cell populations. For visualisation, the uniform manifold approximation and projection (UMAP)^22^ techniques were utilised, which were executed with the RunUMAP functions. Major cell types were annotated based on the classic marker genes of each cell type. Each major cell type was subsequently re‐clustered using the same workflow, and the resulting groups were defined as ‘subclusters’ within that cell type.

Cell‐type tissue preference was quantified following the strategy described in the study by Zhang Zemin's team.^23^, ^24^ Briefly, for each cell type/state, we compared its distribution across tissues (blood, adjacent non‐tumour and tumour) and computed a tissue preference score based on the observed versus expected proportions, with statistical significance assessed using the same procedure as previously reported. Detailed definitions and implementation were referenced from the original publication.

Subcluster‐enriched gene sets for enrichment analyses were identified within each corresponding major cell type using Seurat FindMarkers (adjusted *p* value < .05 and avg_log_2_FC > .5). In parallel, subcluster‐specific signature candidates were derived by performing FindMarkers for each subcluster against all remaining cells in the dataset using the same thresholds. Genes satisfying both criteria were retained as the final subcluster signature. Single‐sample enrichment scores of these signatures were computed using the GSVA (v2.0.7) package with the ssGSEA method.^25^ The resulting ssGSEA scores were then evaluated in bulk transcriptomic cohorts for downstream survival analyses, following the same Kaplan–Meier and log‐rank methods described above.

Pseudotime analysis was performed using Monocle3 (v1.4.26) on the selected cell subset.^26^ To enable Monocle3 analysis, the Seurat object was first converted into a cell_data_set, followed by UMAP projection and trajectory construction using learn_graph. Cells were ordered along the trajectory using order_cells, with the root state defined by the cell population representing the earliest differentiation stage. Pseudotime‐associated genes were identified using graph_test, and genes with significant association were grouped into modules using Monocle3 gene module clustering. Module‐level expression dynamics along pseudotime were visualised as heatmaps with cells ordered from early to late pseudotime. Genes showing similar dynamic changes across pseudotime were grouped into five clusters (C1–C5) and presented as a pseudotime heatmap.

To infer intercellular interaction, the R package CellChat (v1.6.1)^27^ was used. The raw count expression matrix and subcluster annotations were used as input to infer the interactions between subclusters.

### 2.8 Spatial transcriptomics analysis

Stereo‐seq data from HCC01 and HCC03 at bin100 resolution were used for spatial analyses. Spatial enrichment of DPT cells, M2 macrophages and capillary‐associated endothelial cells (ECs) was evaluated on the spatial assay. For each cell population, signature genes derived from the previously defined signatures were used to calculate a spot‐level score. Specifically, the expression values of signature genes detected in the dataset were summed for each spot to obtain the corresponding signature score.

For each signature, spots with scores above the 97.5th percentile were defined as high‐enrichment spots. To extend these high‐enrichment spots to local niches, neighbouring spots were identified using the spatial coordinates in the Stereo‐seq object. In the bin100 Stereo‐seq data, a neighbourhood distance of 1 was applied, and each high‐enrichment spot together with its surrounding spots within the local 3×3 coordinate window was considered part of the corresponding niche. After merging all neighbouring spots and removing duplicates, binary labels were assigned to define DPT‐high, capillaryEC‐high and Macrophage_M2‐high niches.^17^

To assess the spatial relationship between DPT cells and the other two niches, DPT signature scores were compared between capillaryEC‐high and capillaryEC‐low spots, as well as between Macrophage_M2‐high and Macrophage_M2‐low spots. Only spots with DPT scores greater than 0 were included in these comparisons. Statistical significance was evaluated using the Wilcoxon rank‐sum test.

### 2.9 Flow cytometric analysis and sorting of DPT cells

Paired peripheral blood and tumour tissue samples from three patients with HCC were used for analysis of DPT cell distribution. In addition, peripheral blood samples from another four patients were used for flow cytometric analysis of PD‐1 expression in DPT cells and CD8^+^ T cells. Among these four patients, one patient with a relatively high proportion of DPT cells was further subjected to flow cytometric sorting to obtain DPT cells for subsequent expansion and in vitro assays. Single‐cell suspensions from tumour tissues were prepared as described above. In peripheral blood samples, erythrocytes were eliminated with 1× BD Pharm Lyse buffer (555899; BD Biosciences, San Jose, CA, USA), and the nucleated cell fraction was subsequently washed and prepared for later staining.

For DPT cell identification and sorting, cells were stained with LIVE/DEAD Fixable Aqua Dead Cell Stain (L34966A; Thermo Fisher Scientific, Waltham, MA, USA), a CD3‐FITC/CD4‐PE/CD45‐PerCP fluorescent monoclonal antibody kit (Z6410001; Beijing Kuangbo Biotechnology Co., Ltd., Beijing, China) and APC anti‐human CD8 antibody (980904; BioLegend, San Diego, CA, USA). For PD‐1 detection, BV421 anti‐human CD279 (PD‐1) antibody (562516; BD Biosciences) was added to the staining panel. After staining, cells were washed and analysed or sorted by flow cytometry. DPT cells were defined as viable CD45^+^CD3^+^CD4^+^CD8^+^ cells, and CD8+ T cells were defined as viable CD45^+^CD3^+^CD8^+^CD4− cells. The percentage of PD‐1+ cells in DPT cells and CD8^+^ T cells was then analysed by flow cytometry. Detailed methods for the related cell‐based experiments are provided in the Supporting Information, Methods.

### 2.10 Statistical analyses

Unless otherwise indicated, statistical evaluation was carried out in GraphPad Prism version 10.0. For two‐group comparisons, paired data were examined with the Wilcoxon matched‐pairs signed‐rank test, whereas independent samples were evaluated with the Mann–Whitney *U* test. Differences across three or more groups were assessed by the Kruskal–Wallis test. Categorical data were examined with either the chi‐square test or Fisher's exact test, depending on data distribution. quantitative PCR (qPCR) experiments involving multiple genes and treatment conditions were evaluated by two‐way ANOVA. When multiple comparisons were involved, false discovery rate control was applied with the Benjamini‐Hochberg procedure. Associations between continuous variables were determined by Spearman rank correlation. Cox proportional hazards models are described in the relevant sections above. All tests were two‐tailed, and statistical significance was defined as *p* < .05.

## 3 RESULTS

### 3.1 The tumour immune landscape in HCC

In the Xiangya cohort, long‐term survivors were defined as a follow‐up time more than 2 years. In the Fudan–Huashan cohort, given that the follow‐up duration for all cases was less than 2 years, patients who experienced death events during follow‐up were assigned to the short‐term survival group. To investigate the differences in immune, stromal, and cancer cell composition between long‐ and short‐term survivors, we analysed the transcriptome data of two cohorts: the Xiangya HCC cohort and the Fudan–Huashan HCC cohort. We calculated the ESTIMATE score for each tumour sample and then grouped all the cases into long‐ and short‐term survival groups. The ESTIMATE scores of each case were provided in Tables S8 and S9. The ESTIMATE scores, including TumorPurity, StromalScore and ImmuneScore, were not significantly different between the two groups, neither in the Xiangya cohort (Figure S1A) nor in the Fudan–Huashan cohort (Figure S1B). These findings suggest that bulk‐level immune/stromal proportions, as estimated by ESTIMATE, are insufficient to explain the prognostic divergence between the two survival groups. Therefore, we turn to the tumour's and adjacent liver's single‐cell landscape based on mass cytometry data in the Xiangya cohort. All data were cleaned to get single live cells for further analysis (Figure S1C).

**FIGURE 1:**
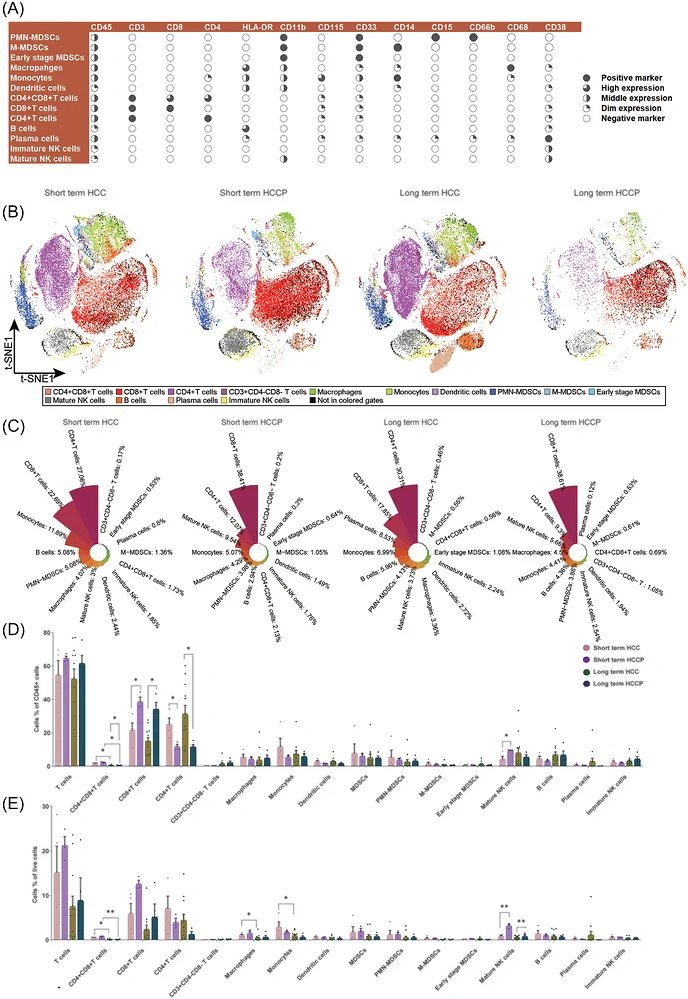
Composition and differential analysis of immune cell populations between short‐ and long‐term survivors. (A) Immune cell markers were curated using the Human Protein Atlas and published literature. (B) t‐SNE visualisation of grouped samples from the Xiangya cohort. (C) Sunburst plot showing the proportions of major immune cell populations across different groups in the Xiangya cohort. (D and E) Relative frequencies of major immune cell populations across different groups in the Xiangya cohort. Percentages within total immune cells (D) and within all live cells (E) are shown. ns, not significant. *p < .05.

The tumour immune microenvironment of short‐ and long‐term survivors in the Xiangya cohort showed heterogeneity. We systematically collected immune cell markers from publications and the Human Protein Atlas website (Figure 1A), and we categorised CD45^+^ cells into 14 groups based on a combination of these markers (Figure S1C,D). Using t‐SNE, we separately visualised the immune cell groups in the tumours and adjacent tissues of short‐ and long‐term survivors (Figure 1B). The percentages of immune cell groups within CD45^+^ cells were presented utilising the sunburst plot, separately in the tumours and adjacent tissues of short‐ and long‐term survivors (Figure 1C,D). Comparative analysis of immune cell composition in cancerous and adjacent tissues revealed a significant increase in the proportion of total CD4^+^ T cells among CD45^+^ cells in tumours. Conversely, the proportion of total CD8^+^ T cells among CD45^+^ cells exhibited a decrease in cancerous tissues (Figure 1D,E and Table S10). The proportions of CD4^+^ T cells and CD8^+^ T cells in tumour and adjacent non‐tumour tissues did not show significant differences between short‐term and long‐term survivors, whether calculated as percentages of total live cells or CD45^+^ immune cells (Figure 1D,E).

### 3.2 Tumour‐infiltrating DPT cells roleplay as poor prognosis indicators and express exhausted immune markers

**FIGURE 2:**
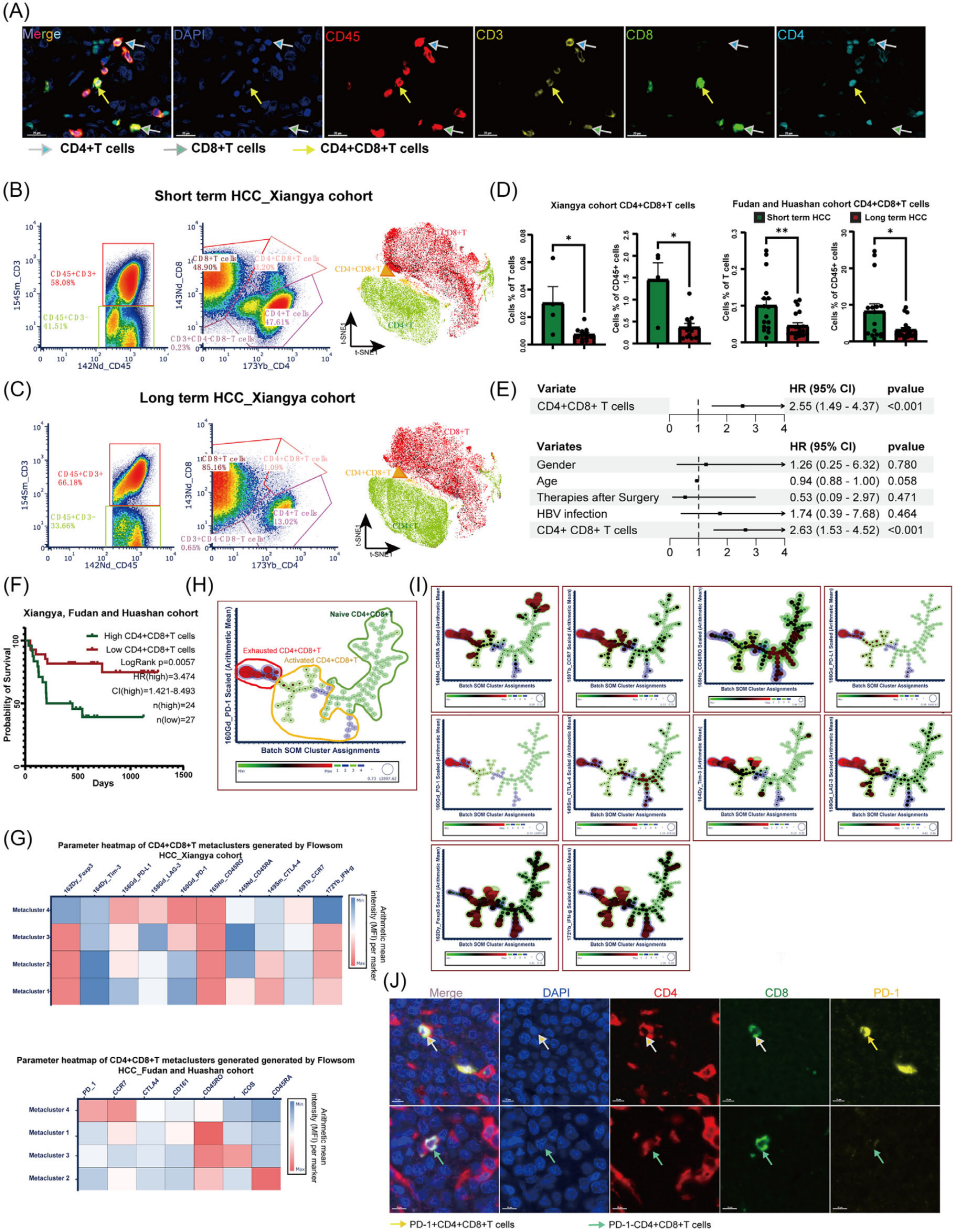
Tumour‐infiltrating DPT cells are a risk factor for patients with HCC. (A) Representative mIHC images showing DPT cells in tumour tissue. (B and C) Gating strategy for DPT cells in tumours and t‐SNE visualisation of tumour‐infiltrating T‐cell composition from short‐term (B) and long‐term (C) survivors in the Xiangya cohort. (D) Comparison of DPT cell frequencies between short‐ and long‐term survivors in the Xiangya and Fudan–Huashan cohorts. (E) Univariate and multivariate Cox proportional hazards regression in the merged Xiangya and Fudan–Huashan cohort evaluating the prognostic impact of DPT cells. (F) Kaplan–Meier survival analysis with log‐rank test in the merged Xiangya and Fudan–Huashan cohort evaluating the prognostic impact of DPT cells. (G) Heatmap showing immune checkpoint distribution across four meta‐clusters generated by the FlowSOM algorithm in the Xiangya and Fudan–Huashan cohorts. (H) Minimum spanning tree (MST) of DPT cells from Fudan–Huashan cohort, showing three DPT subsets. (I) MST of DPT cells from Fudan–Huashan cohort showing the distribution of key markers. (J) Representative mIHC images of PD‐1^bright^ CTLA4^dim^ and PD‐1^dim^ CTLA4^bright^ DPT cells. ns, not significant. *p < .05. **p < .01. CI, confidence interval; HR, hazard ratio.

Among all the cell types, we identified a substantial elevation in the proportions of DPT cells in both cancerous and adjacent tissues of short‐term survivors, surpassing those of long‐term survivors, either within CD45^+^ cells or overall live cells (Figure 1D,E and Table S10). To visualise the in situ presence and localisation of DPT cells, we first used multiplexed immunochemistry (mIHC) to visualise DPT cells in the cancerous tissue (Figure 2A). To get deeper insight into the tumour‐infiltrating DPT cells, we quantified the frequency of DPT cells among tumour‐infiltrating T cells, separately in short‐ and long‐term survivors of two independent cohorts (Figures 2B,C and S2A,B). The percentage of DPT cells among tumour‐infiltrating CD45^+^ cells and T cells was higher in short‐term survivors than in long‐term survivors (Figure 2D). Kaplan–Meier survival analysis, log‐rank tests, univariate Cox hazard analysis and multivariate Cox hazard analysis further showed an adverse prognostic association of tumour‐infiltrating DPT cells, with hazard ratios higher than two and *p* values lower than  .01 (Figure 2E,F). Moreover, we substantiated the widespread presence of DPT cells in mice by employing four syngeneic mouse models featuring TIBx, RIL‐175, HEP53.4 and Hepa1‐6 cells (Figure S2C). Consistently, mIHC of tumour sections further detected DPT cells in both orthotopic and spontaneous murine HCC models (Figure S2D).

To define the subgroups of tumour‐infiltrating DPT cells, we used the FlowSOM algorithm to generate four meta‐clusters and visualise the immune marker expression of each meta‐cluster (Figure 2G). The four meta‐clusters in the Xiangya cohort had three expression patterns. One was characterised by positive LAG‐3, PD‐1, CCR7, CD45RA and negative IFNG, consistent with an exhausted‐like phenotype. One was marked with negative PD‐1, TIM‐3, LAG‐3 and positive CD45RA, suggesting its naive state. The others had relatively low CD45RA, PD‐1, LAG‐3, TIM‐3 and high CD45RO and IFNG, indicating their functionally activated state (Figure 2G). Similar expression patterns existed in the Fudan–Huashan cohort (Figure 2G). Therefore, the tumour‐infiltrating DPT cells comprised three groups: naive, activated and exhausted (Figure 2H). The minimum spanning tree of DPT cells showed that inhibitory receptors, including PD‐1, TIM‐3 and LAG‐3, were progressively up‐regulated from naive to activated and exhausted states (Figure 2I). The mIHC on human tumour tissues further visualised PD‐1^+^ and PD‐1^−^ DPT cells (Figure 2J).

### 3.3 DPT cells have tissue‐preference and niche‐preference, cross‐talking with capillary‐associated endothelial cells and M2 macrophages

**FIGURE 3:**
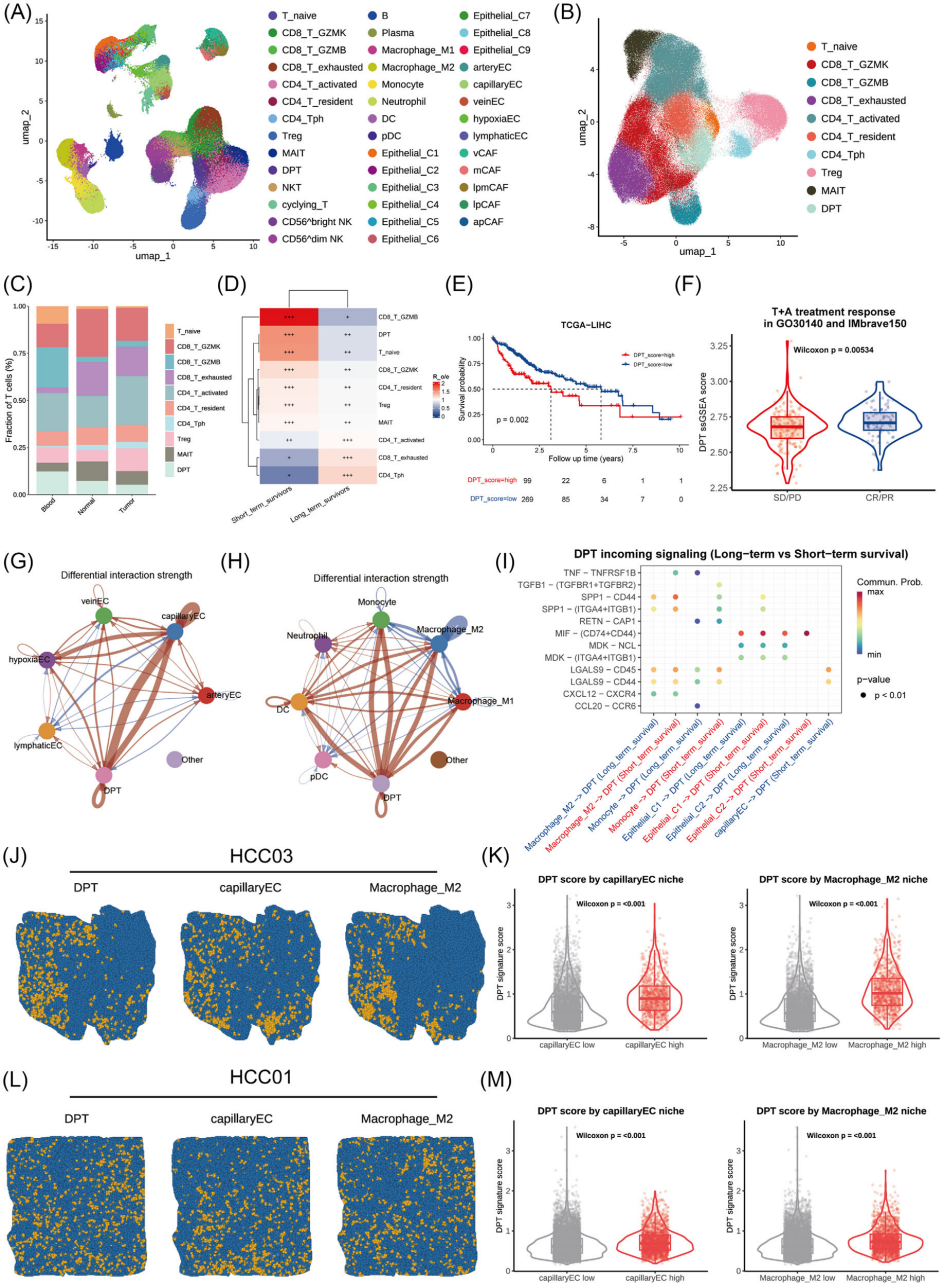
Single‐cell validation reveals the malignant role of DPT cells in HCC. (A) UMAP visualisation of single‐cell annotations in the PRJCA007744 dataset. (B) UMAP visualisation of annotated T‐cell subgroups. (C) Stacked bar plot showing T‐cell subset proportions across blood, adjacent non‐tumour tissue and tumour in the PRJCA007744 dataset. (D) Relative odds enrichment heatmap showing tissue preference of T‐cell subset in short‐term versus long‐term survival groups in the PRJCA007744 dataset. (E) TCGA‐LIHC Kaplan–Meier survival analysis stratified by DPT signature ssGSEA score. (F) DPT signature ssGSEA score versus atezolizumab plus bevacizumab (T+A) response in GO30140 and IMbrave150 (SD/PD vs. CR/PR) cohort. (G and H) Compartment‐level CellChat differential interaction networks (short‐ and long‐term survivors) in the PRJCA007744 dataset. Edge width indicates differential interaction strength. Red indicates stronger interactions in short‐term survivors, and blue indicates stronger interactions in long‐term survivors. (I) CellChat bubble plots of DPT incoming signalling in the PRJCA007744 dataset. (J) Spatial distribution of DPT cells, capillary‐associated ECs and M2 macrophages in the HCC03 sample profiled by Stereo‐seq at bin100 resolution, accession CNP0004497. Bins enriched for each cell type are highlighted in yellow. (K) Box plot showing DPT signature expression in bins with different enrichment patterns of capillary‐associated EC or M2 macrophages in HCC03, accession CNP0004497. (L) Spatial distribution of DPT cells, capillary‐associated ECs and M2 macrophages in the HCC01 sample profiled by Stereo‐seq at bin100 resolution, accession CNP0004497. Bins enriched for each cell type are highlighted in yellow. (M) Box plot showing DPT signature expression in bins with different enrichment patterns of capillary‐associated ECs or M2 macrophages in HCC01, accession CNP0004497. EC, endothelial cell; ssGSEA, single‐sample gene set enrichment analysis.

In the single‐cell cohort, patients with a follow‐up duration of more than 2 years were assigned to the long‐term survivor group, whereas patients who died within 2 years were assigned to the short‐term survivor group. To further validate DPT cells and characterise their functional heterogeneity, we performed single‐cell transcriptomic profiling and downstream integrative analyses (Figures S3A–G and S4A,B). In total, the dataset was annotated into 41 clusters spanning major cellular lineages (Figures 3A and S3A–G).

Focusing on the T‐cell compartment, UMAP revealed distinct T‐cell subpopulations (Figures 3B and S3H). DPT cells were most abundant in peripheral blood compared with tissue compartments (Figure 3C). Two complementary tissue‐preference scoring strategies consistently supported a preferential distribution of DPT cells in blood (Figure S4C,D). Nevertheless, tissue‐preference analyses further showed that, relative to long‐term survivors, DPT cells displayed a stronger tendency to distribute in tissues from short‐term survivors (Figures 3D and S4E). Pathway enrichment analysis of DPT cell differentially expressed genes highlighted immune checkpoint‐related programs among the significantly enriched pathways (Figure S4F). Consistently, DPT cells exhibited detectable expression of immune checkpoint molecules (Figure S4G). In external validation cohorts, higher DPT scores were consistently associated with poorer prognosis in TCGA‐LIHC, ICGC and CHCC (Figures 3E and S4H,I). In addition, DPT scores were associated with response to atezolizumab plus bevacizumab therapy (Figure 3F).

We next interrogated the intercellular communication context of DPT cells across cellular subtypes (Figures 3G,H and S4J). Compared with long‐term survivors, DPT cells in short‐term survivors received stronger incoming signals from M2 macrophages, monocytes, epithelial subclusters and capillary‐associated ECs, and exhibited stronger outgoing signalling towards CD56^dim^ NK cells, M2 macrophages, monocytes, dendritic cells and capillary ECs (Figures 3I and S4K). To further examine spatial relationships, we analysed spatial transcriptomic sections and evaluated the spatial association between DPT cells, M2 macrophages and capillary‐associated ECs. Based on cell‐type signature expression, the top 2.5% of spots and their neighbouring spots were defined as enrichment regions (Figure 3J,L). DPT scores were higher in and around both capillary‐associated endothelial‐enriched regions and M2 macrophage‐enriched regions, supporting co‐localisation of DPT cells with these components within the TME (Figure 3K,M).

To resolve DPT heterogeneity, DPT cells were further classified into three major states, namely, naïve, activated and exhausted subsets (Figure S5A–C). The naïve DPT subset preferentially distributed in blood and was relatively enriched in long‐term survivors, whereas the exhausted DPT subset showed the opposite pattern, with a higher tendency to distribute in tissues and in short‐term survivors (Figure S5D–G). Pseudotime analysis further supported the inferred state transition and the above state annotations (Figure S5B,C). In addition, KEGG enrichment analysis of differentially expressed genes across DPT subclusters revealed distinct pathway preferences (Figure S5H): activated DPT was predominantly enriched in MAPK and TNF signalling pathways, whereas exhausted DPT showed preferential enrichment in mTOR signalling and apoptosis pathways, collectively reinforcing the functional divergence between these states.

We performed additional subgroup analyses in patients with HBV infection (Figure S5I,J). In both the Xiangya and Fudan cohorts, a higher proportion of DPT cells among T cells was consistently associated with shorter survival, whereas the proportion of DPT cells among CD45^+^ cells was associated with poorer outcomes only in the Xiangya cohort. In the TCGA cohort, we further conducted subgroup analyses of the DPT signature according to HBV infection status, tumour stage, HCV infection status and liver fibrosis. The corresponding results are presented in Figure S5K–O.

We also evaluated tissue‐distribution preferences across all other cellular subclusters in the single‐cell dataset (Figure S6). We found that CD56^dim^ NK cells, capillary ECs and epithelial C8 subsets were enriched in tumour tissue of short‐term survivors.

### 3.4 Tumour‐infiltrating CD8^+^ T cells have a diminished frequency in short‐term survivors

**FIGURE 4:**
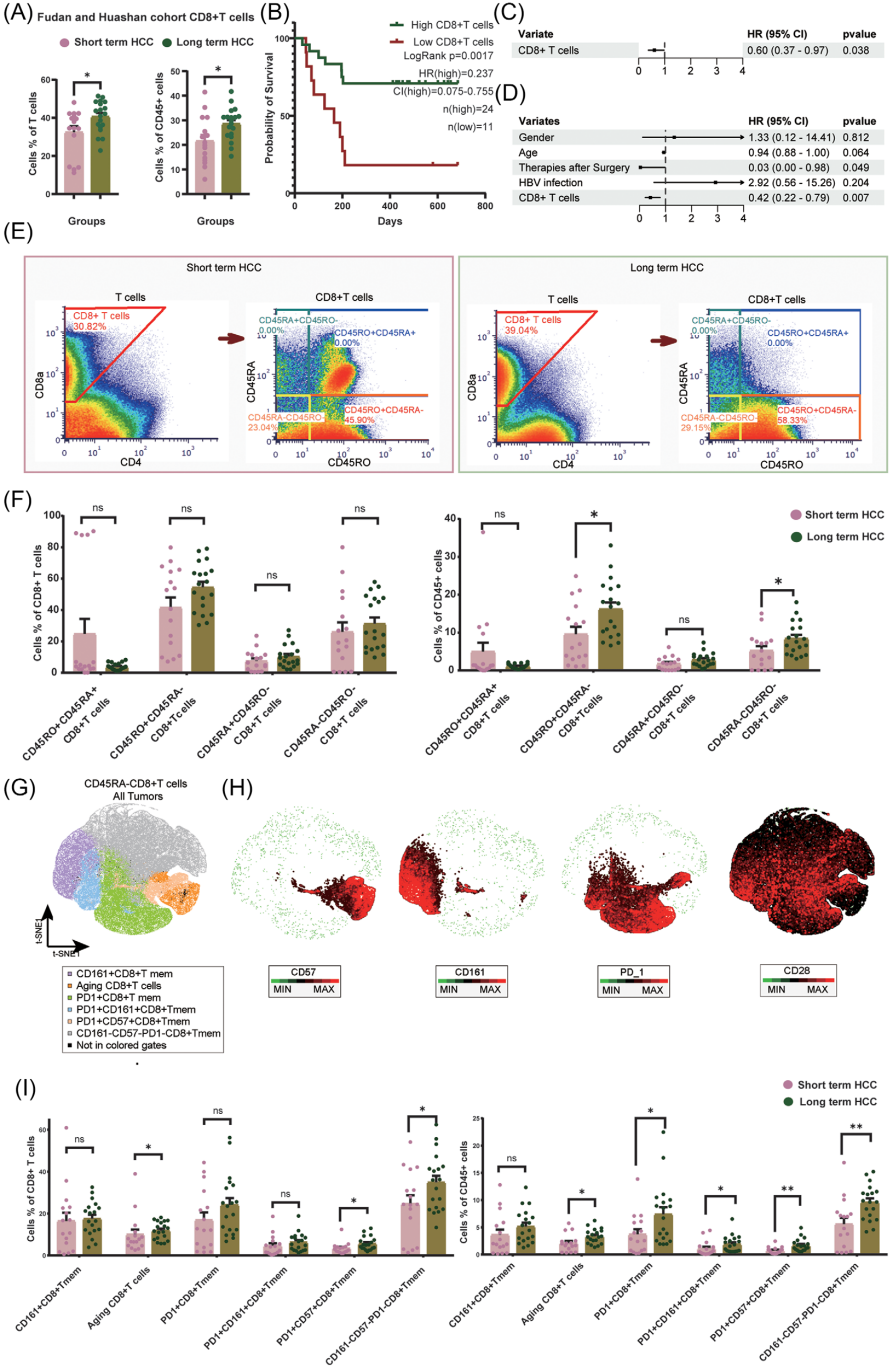
Tumour‐infiltrating CD8^+^ T cells are protective factors for patients with HCC. (A) Relative frequencies of tumour‐infiltrating CD8^+^ T cells between short‐ and long‐term survivors in the Fudan–Huashan cohort. Percentages within total T cells and within CD45^+^ cells are shown. (B) Kaplan–Meier survival analysis with log‐rank test in the Fudan–Huashan cohort evaluating the prognostic significance of tumour‐infiltrating CD8^+^ T cells. (C) Univariate Cox proportional hazards regression in the Fudan–Huashan cohort evaluating the prognostic impact of tumour‐infiltrating CD8^+^ T cells. (D) Multivariate Cox proportional hazards regression in the Fudan–Huashan cohort evaluating the prognostic impact of tumour‐infiltrating CD8^+^ T cells. (E) Gating strategy for CD45RA^+^CD45RO^+^, CD45RA^+^CD45RO^−^, CD45RA^−^CD45RO^+^ and CD45RA^−^CD45RO^−^ CD8^+^ T cells in tumours from patients with short‐ and long‐term survivors in the Fudan–Huashan cohort. (F) Relative frequencies of tumour‐infiltrating CD45RA^+^CD45RO^+^, CD45RA^+^CD45RO^−^, CD45RA^−^CD45RO^+^ and CD45RA^−^CD45RO^−^ CD8^+^ T cells between short‐ and long‐term survivors in the Fudan–Huashan cohort. Percentages within total CD8^+^ T cells and within CD45^+^ cells are shown. (G) t‐SNE visualisation of tumour‐infiltrating CD45RA^−^ CD8^+^ T cells (CD8^+^ Tmem) across all survival groups from the Fudan–Huashan cohort. (H) t‐SNE visualisation showing the distribution of CD57, CD161, PD‐1 and CD28 across all survival groups from the Fudan–Huashan cohort. (I) Relative frequencies of tumour‐infiltrating CD8^+^ Tmem subsets between short‐ and long‐term survivors from the Fudan–Huashan cohort. Percentages within total CD8^+^ T cells and within CD45^+^ cells are shown. ns, not significant. *p < .05. CD8^+^ Tmem, CD8^+^ memory T cells; CI, confidence interval; HR, hazard ratio.

To investigate the prognostic implications of CD8^+^ T cells and their subsets, we analysed the mass cytometry data from the Fudan–Huashan cohort, which encompassed diverse T subset markers (Table S11). The frequency of CD8^+^ T cells among tumour‐infiltrating CD45^+^ immune cells was lower in short‐term survivors than in long‐term survivors (Figure 4A). Subsequently, we conducted Kaplan–Meier survival analysis, log‐rank tests and univariate Cox hazard analysis, with the outcomes consistently reaffirming the protective influence of CD8^+^ T cells on prognosis (Figure 4B,C). Notably, the results from multivariate Cox hazard analysis underscored the capacity of CD8^+^ T cells as a robust independent protective factor for overall survival (Figure 4D).

To identify dominant CD8^+^ T cell subsets influencing overall survival, we classified cells into four subgroups based on CD45RA and CD45RO expression (Figure 4E). No significant differences existed in CD45RO^+^CD45RA^+^CD8^+^ T cells and CD45RO^−^CD45RA^+^CD8^+^ T cells between the short‐ and long‐term survivors (Figure 4F). However, it was evident that both CD45RO^+^CD45RA^−^CD8^+^ T cells and CD45RO^−^CD45RA^−^CD8^+^ T cells exhibited a significantly lower frequency within short‐term survivors’ tumours than in long‐term survivors (Figure 4F). Kaplan–Meier survival analysis, log‐rank tests and univariate Cox hazard analysis further confirmed their prognostic significance (Figure S7A,B). According to previous studies, the negative expression of CD45RA represented the memory state of CD8^+^ T cells. We classified memory CD8^+^ T cells into six subsets according to the expression of CD161, CD57, PD‐1 and CD28 (Figure 4G,H). Most of the subsets significantly diminished within the tumours of the short‐term survivors. Moreover, most of them had a protective significance, indicating that the total amount instead of subsets of memory CD8^+^T cells impacted overall survival (Figures 4I and S7A,B).

### 3.5| Tumour‐infiltrating CD45RA^+^CD4^+^ T conventional cells are enriched in short‐term survivors and show an adverse prognostic association, while CD45RA^−^CD4^+^ T conventional cells display the opposite pattern

**FIGURE 5:**
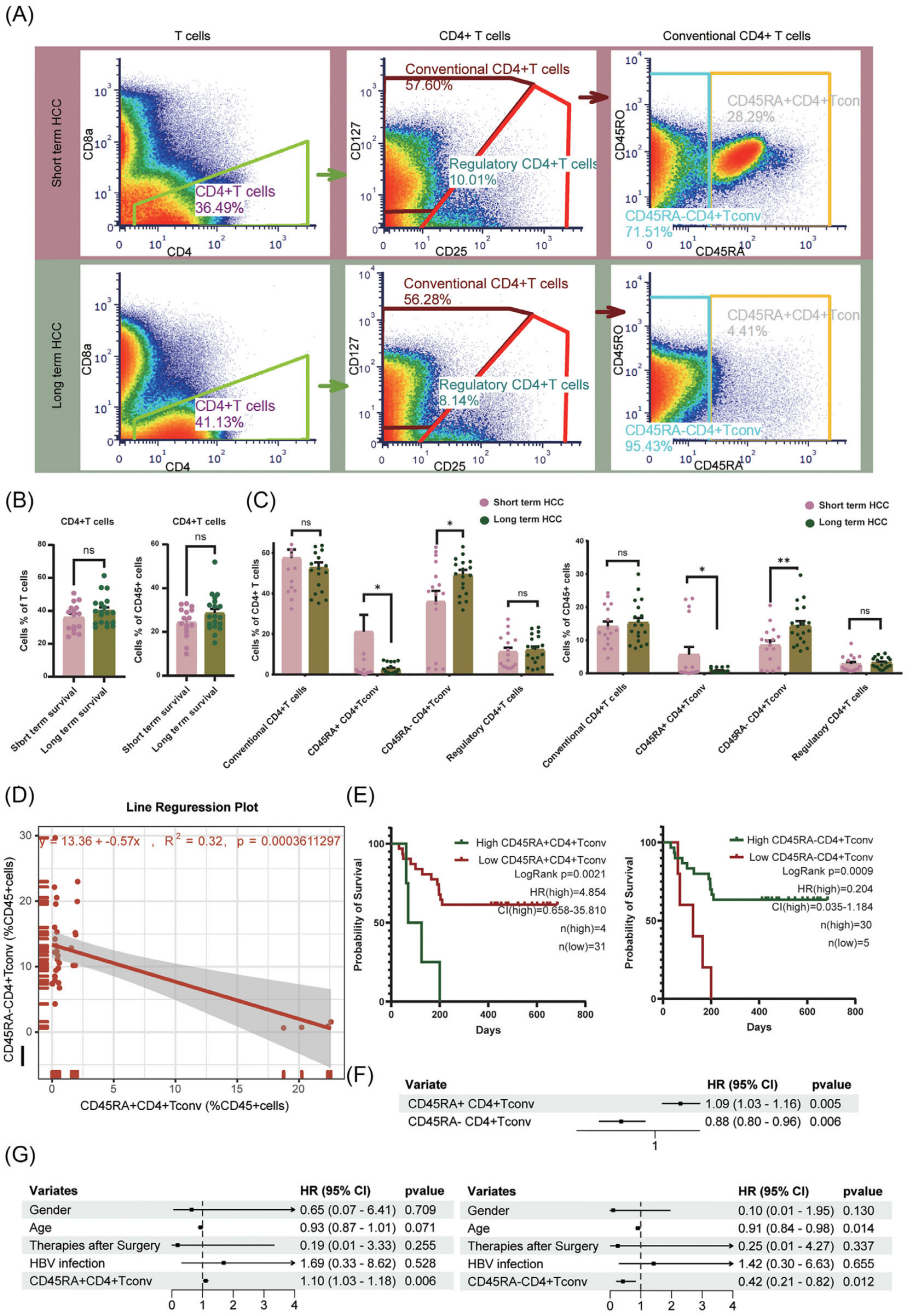
Tumour‐infiltrating CD45RA^+^CD4^+^ conventional T cells are risk factors, whereas CD45RA^−^ CD4^+^ conventional T cells are protective factors for patients with HCC. (A) Gating strategy for CD4^+^ T cells in tumours from patients with short‐ and long‐term survivors from the Fudan–Huashan cohort. (B) Relative frequencies of tumour‐infiltrating CD4^+^ T cells between short‐ and long‐term survivors. Percentages within total T cells and within CD45^+^ cells are shown. (C) Comparative analysis of tumour‐infiltrating CD4^+^ T‐cell subset frequencies between short‐ and long‐term survivors. Percentages within total CD4^+^ T cells and within CD45^+^ cells are shown. (D) Linear regression plot showing the correlation between CD45RA^+^CD4^+^ and CD45RA^−^ CD4^+^ conventional T cells. (E) Kaplan–Meier survival analysis with log‐rank test in the Fudan–Huashan cohort evaluating the prognostic significance of CD45RA^+^CD4^+^ T cells and CD45RA^−^CD4^+^ Tconv cells. (F) Univariate Cox proportional hazards regression in the Fudan–Huashan cohort evaluating the prognostic significance of CD45RA^+^CD4^+^ T cells and CD45RA^−^CD4^+^ Tconv cells. (G) Multivariate Cox proportional hazards regression in the Fudan–Huashan cohort evaluating the prognostic significance of CD45RA^+^CD4^+^ T cells and CD45RA^−^CD4^+^ Tconv cells. ns, not significant. *p < .05. CI, confidence interval; HR, hazard ratio.

We stratified CD4^+^ T cells from the Fudan–Huashan cohort into conventional CD4^+^ T cells (CD4^+^ Tconv) and regulatory CD4^+^ T cells based on the expression of CD127 and CD25. We further divided CD4^+^ Tconv cells into CD45RA^+^CD4^+^ and CD45RA^−^CD4^+^ Tconv cells (Figure 5A). While there were no discernible differences in the infiltration of CD4^+^ T cells between the tumours of short‐ and long‐term survivors, we did observe a higher frequency of CD45RA^+^CD4^+^ Tconv cells and a lower frequency of CD45RA^−^CD4^+^ Tconv cells in the tumours of short‐term survivors compared with their long‐term counterparts (Figure 5B,C). The percentage of CD45RA^+^CD4^+^ Tconv cells among immune cells exhibited a reverse correlation with that of CD45RA^−^CD4^+^ Tconv cells (Figure 5D). Subsequently, we performed Kaplan–Meier survival analysis, log‐rank tests and univariate Cox hazard analysis, consistently confirming the protective influence of CD45RA^−^CD4^+^ Tconv cells and the unfavourable impact of CD45RA^+^CD4^+^ Tconv cells on prognosis (Figure 5E,F). The multivariate Cox hazard analysis results emphasised the capacity of CD45RA^−^CD4^+^ Tconv cells and CD45RA^+^CD4^+^ Tconv cells as independent predictive factors for overall survival (Figure 5G).

To identify dominant CD45RA^−^CD4^+^ Tconv cell subsets impacting overall survival, we classified cells into four subgroups based on CD161, CD57, CD28 and PD‐1 expression, as depicted in Figure S8A,B. Notably, no significant differences existed in CD45RA^−^CD4^+^ Tconv subsets, except for CD161^+^CD45RA^−^CD4^+^ Tconv cells, indicating the potential of CD161^+^CD45RA^−^CD4^+^ Tconv cells as prognostic factors (Figure S8C). Subsequently, we performed Kaplan–Meier survival analysis, log‐rank tests and univariate Cox hazard analysis, consistently confirming the protective influence of CD161^+^CD45RA^−^CD4^+^ Tconv cells on prognosis (Figure S8D,E). We also observed that CD161 (KLRB1) was highly expressed in mucosal‐associated invariant T (MAIT) (Figure S8F).

### 3.5 HBEGF is a potential risk factor for patients with HCC

**FIGURE 6:**
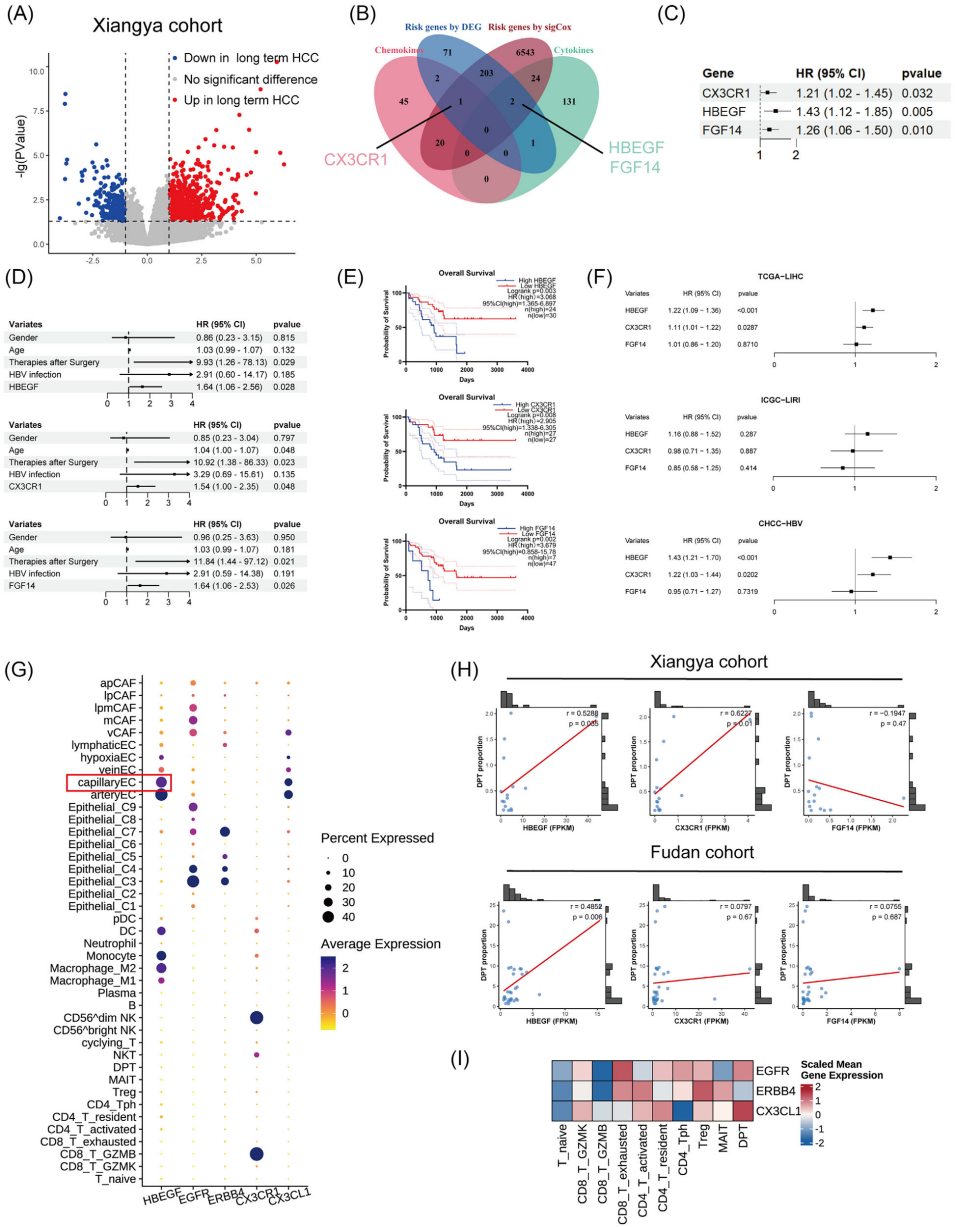
Multi‐cohort transcriptomic and multi‐omics analyses identify HBEGF as potential risk factor in HCC and reveal their subcellular localisation and association with DPT cells. (A) Volcano plot showing differentially expressed genes between patients with short‐ and long‐term survivors in the Xiangya HCC cohort. (B) Intersection of differential gene sets from Xiangya cohort, prognostic gene sets identified through bulk univariate Cox regression and cytokines/chemokines, identifying one chemokine receptor (CX3CR1) and two cytokines (HBEGF and FGF14) with prognostic significance. (C and D) Univariate and multivariate Cox regression analyses for CX3CR1, HBEGF and FGF14 in the Xiangya cohort. (E) Kaplan–Meier survival analysis of CX3CR1, HBEGF and FGF14 in the Xiangya and Fudan cohorts. (F) Univariate Cox regression analysis for HBEGF, CX3CR1 and FGF14 in the TCGA‐LIHC, ICGC‐LIRI and CHCC‐HBV cohort. (G) scRNA‐seq dot plot from the PRJCA007744 dataset showing expression of the HBEGF–EGFR/ERBB4 and CX3CL1–CX3CR1 axes across all cell types. (H) Pearson correlation between DPT proportion and gene expression (FPKM) of CX3CR1, HBEGF and FGF14 in the Xiangya and Fudan cohort. (I) Heatmap of scaled mean expression of HBEGF receptors (EGFR and ERBB4) and the CX3CR1 ligand (CX3CL1) across T‐cell subsets from the PRJCA007744 dataset. ns, not significant. *p < .05. CI, confidence interval; HR, hazard ratio.

In the Xiangya cohort, differential expression analysis identified 280 genes with lower expression and 719 genes with higher expression in the long‐survival group than in the short‐survival group (Figure 6A). Univariate Cox regression further identified 16 protective genes and 6793 risk genes. To prioritise prognosis‐related immune molecules, we intersected the risk genes derived from differential expression and univariate Cox analyses with published gene sets covering cytokines, chemokines and their receptors, immune checkpoints and drug targets. This approach identified one chemokine receptor, CX3CR1, and two cytokines, HBEGF and FGF14, as candidate risk‐associated molecules (Figure 6B). Their associations with prognosis are summarised by the univariate and multivariable Cox regression forest plots shown in Figure 6C,D. Patients with high expression of HBEGF, CX3CR1 and FGF14 was associated with shorter overall survival in the Xiangya cohort and the Fudan and Huashan cohort (Figure 6E). In univariate Cox regression analyses across three independent cohorts (TCGA, ICGC and CHCC), CX3CR1 and HBEGF consistently emerged as risk factors in both the TCGA and CHCC cohorts, whereas FGF14 did not show sufficient evidence to be considered a risk factor in any of the three cohorts (Figure 6F).

We further interrogated HBEGF and CX3CR1 together with their cognate ligand–receptor pairs. At the single‐cell level, HBEGF expression was mainly detected in endothelial and myeloid compartments, with prominent signals in capillary ECs, artery ECs, M2 macrophages and monocytes, whereas CX3CR1 was preferentially enriched in GZMB^+^ CD8^+^ T cells and CD56^dim^ NK cells (Figure 6G). Correlation analyses showed that HBEGF was significantly and positively associated with the proportion of DPT cells in both the Xiangya and Fudan cohorts, while CX3CR1 exhibited a positive correlation with DPT abundance only in the Xiangya cohort (Figure 6H). Notably, CX3CL1 (the ligand for CX3CR1) and EGFR (the receptor for HBEGF) were relatively enriched in DPT cells compared with other T‐cell subsets (Figure 6I).

### 3.6 CD45^+^EpCAM^+^ and CD45^+^α‐SMA^+^ cell clusters have a higher frequency in the tumour of short‐term survivors and are risk factors for HCC

**FIGURE 7:**
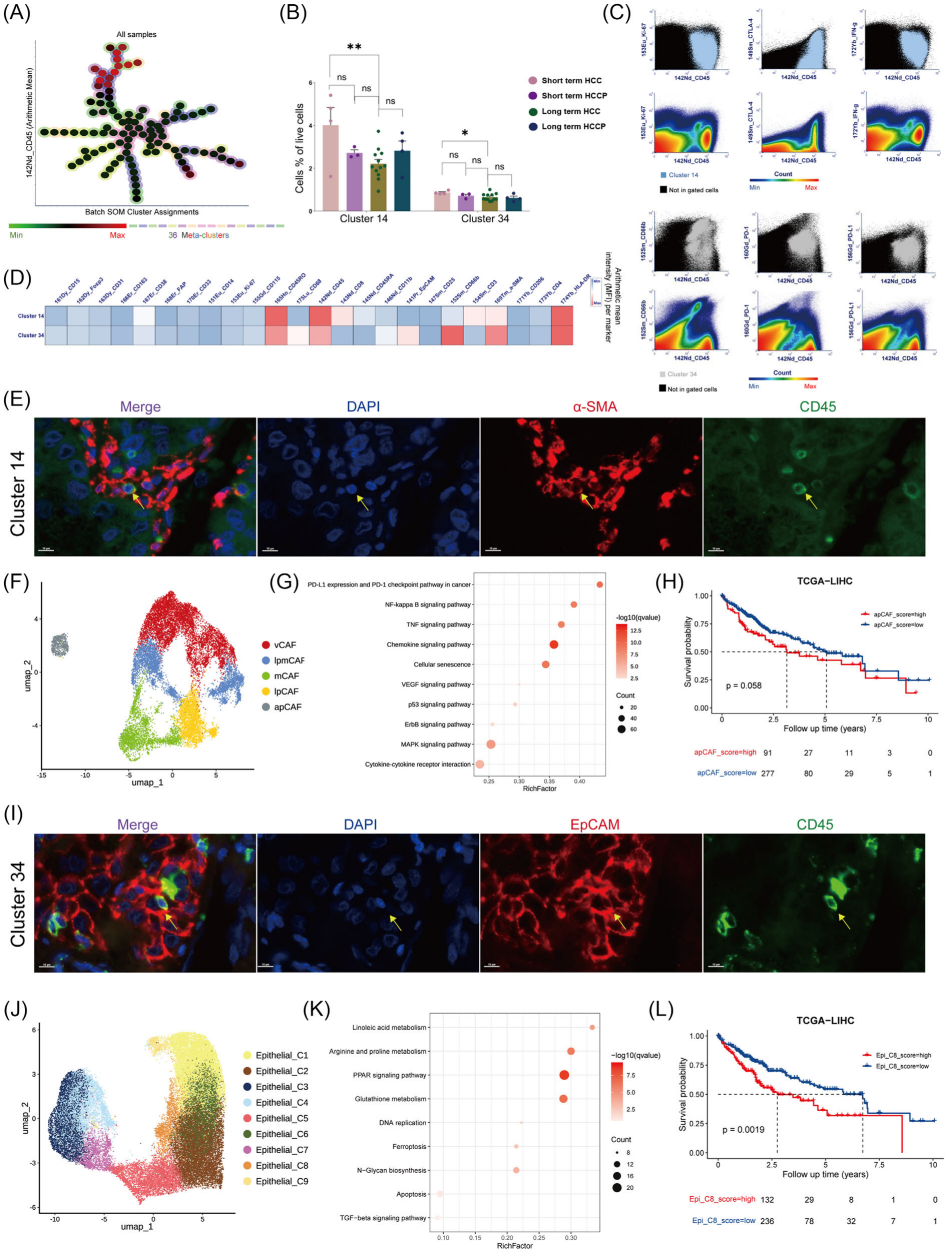
Increased frequency of cell groups co‐expressing immune, stromal, and cancer cell markers in tumours from short‐term survivors is associated with poor prognosis in HCC. (A) FlowSOM clustering of all CyTOF data in the Xiangya cohort. (B) Parameter heatmap of all clusters. (C) Colour dot plots and density plots showing expression of CD45, CD31, FAP, alpha‐SMA, EpCAM and Glypican3 in clusters 14 and 34 in the Xiangya cohort. (D) Parameter heatmap showing the distribution of cell markers in clusters 14 and 34 in the Xiangya cohort. (E) Representative mIHC images of α‐SMA^+^ CD45^+^ cells. (F) UMAP visualisation of annotated cancer‐associated fibroblast subclusters from the PRJCA007744 dataset. (G) Bubble plot of KEGG pathway enrichment for differentially expressed genes in apCAF from the PRJCA007744 dataset. (H) TCGA‐LIHC Kaplan–Meier survival analysis stratified by apCAF signature ssGSEA score. (I) Representative mIHC images of EpCAM^+^ CD45^+^ cells. (J) UMAP visualisation of annotated epithelial cell subclusters from the PRJCA007744 dataset. (K.) Bubble plot of KEGG pathway enrichment for differentially expressed genes in Epithelial_C8 from the PRJCA007744 dataset. (L) TCGA‐LIHC Kaplan–Meier survival analysis stratified by Epithelial_C8 signature ssGSEA score. ns, not significant. *p < .05.

While the prognostic significance of immune cells is well established, we then performed comprehensive profiling of all cell types to uncover previously unrecognised cell clusters. FlowSOM algorithm clustered cells into 36 meta‐clusters (Figure 7A and Table S12). The frequencies of Cluster 14 and Cluster 34 differed significantly between short‐ and long‐term survivors (Figure 7B). The parameter heatmap in Figure S9A,B visualised the expression of CD31, FAP, CD45, Glypican3, EpCAM and α‐SMA in each cluster. Cluster 34 was positive for CD45, α‐SMA, and Ep‐CAM, whereas Cluster 14 was positive for CD45 and α‐SMA (Figure 7C,D). To identify the cell identity of Cluster 14 and Cluster 34, we compared the expression levels of various immune cell markers and checkpoints listed in Table S5. Cluster 14 expressed a high level of CD45, Ki‐67, CTLA4 and IFN‐γ whereas Cluster 34 expressed a high CD45, CD66b and PD‐1 level (Figure S9C).

We identified CD45 and α‐SMA double‐positive cells in tumour tissues from patients with HCC (Figure 7E). Single‐cell transcriptomic analysis further revealed an immune‐like cancer‐associated fibroblast (CAF) subset, termed antigen‐presenting CAFs (apCAFs), which showed transcriptional similarity to Cluster 14 (Figure 7F). Pathway enrichment analysis of apCAF differentially expressed genes highlighted immune checkpoint‐related programs (Figure 7G). Clinically, higher apCAF signature scores were associated with poorer prognosis in the ICGC and CHCC cohorts (Figure S9D,E), whereas this association did not reach statistical significance in TCGA (Figure 7H), and apCAF scores were not significantly associated with response to T+A therapy (Figure S9F).

We also detected CD45 and EpCAM double‐positive cells in HCC tumour tissues (Figure 7I). In the single‐cell dataset, the Epithelial_C8 subset exhibited transcriptomic similarity to Cluster 34 (Figures 7J and S3F). Differential expression and pathway enrichment analyses indicated that Epithelial_C8 was enriched for the PPAR signalling pathway (Figure 7K). Across three independent cohorts, higher Epithelial_C8 signature scores were consistently associated with worse prognosis (Figures 7L and S9H,I), whereas no significant association was observed between the Epithelial_C8 score and T+A therapy response (Figure S9J).

### 3.7 Endothelial‐derived HBEGF promotes DPT migration, whereas DPT co‐culture induces pro‐tumour transcriptional changes and migration in tumour cells

**FIGURE 8:**
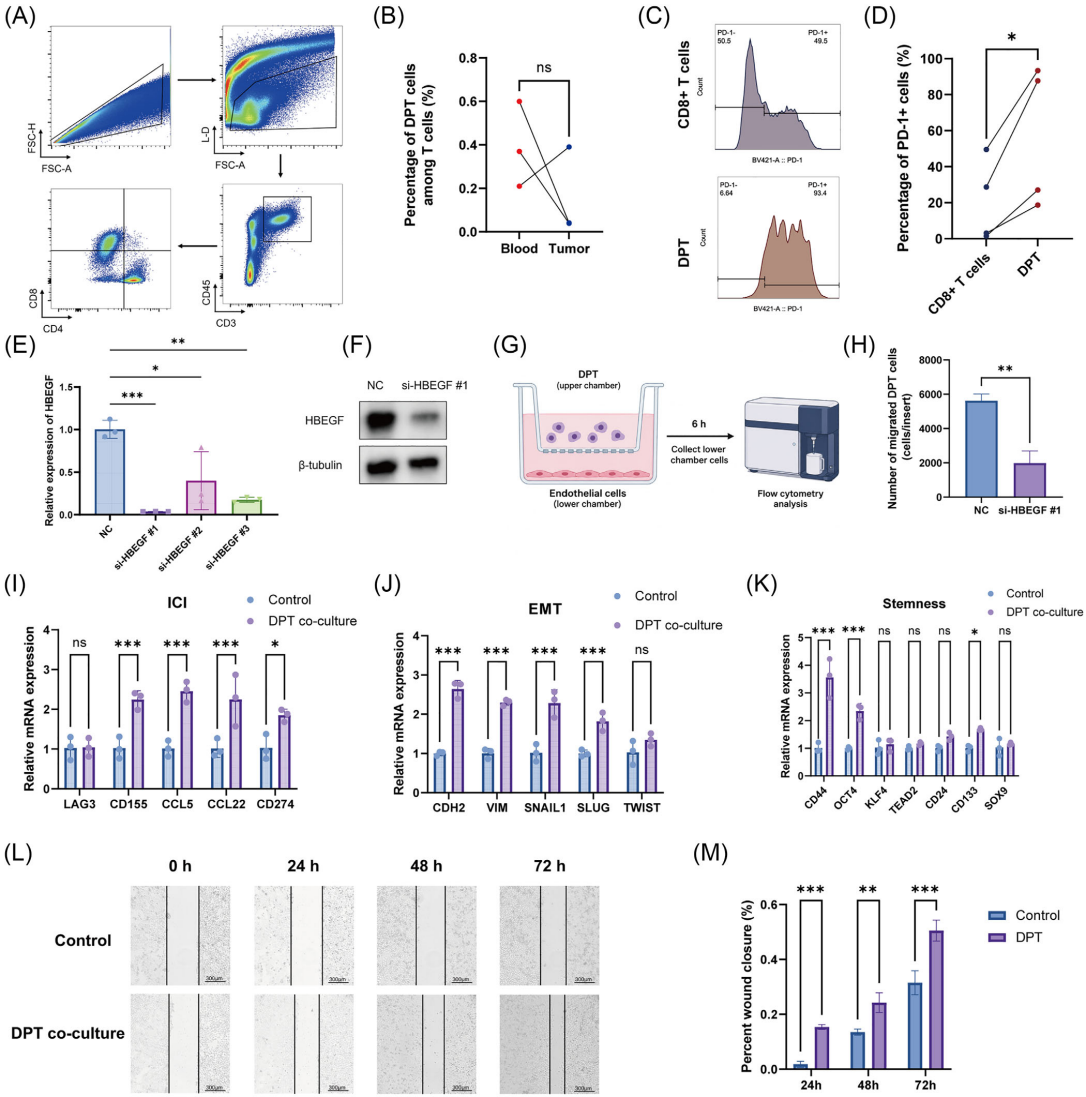
Endothelial‐derived HBEGF promotes DPT migration, and DPT co‐culture induces pro‐tumour phenotypes in tumour cells. (A) Flow cytometric gating strategy for the analysis and sorting of DPT from patients’ peripheral blood or tumour tissues. (B) Paired analysis of the percentage of DPT among T cells in peripheral blood and tumour tissues from three patients. (C) Representative flow cytometry histograms showing PD‐1 expression in CD8^+^ T cells and DPT. (D) Paired analysis of the percentage of PD‐1^+^ cells in CD8^+^ T cells and DPT from four patients. (E) RT‐qPCR analysis of HBEGF expression in AW‐CCH599 endothelial cells after transfection with the indicated siRNAs. (F) Western blot validation of HBEGF knockdown by si‐HBEGF #1. (G) Schematic illustration of the co‐culture assay. (H) Flow cytometric quantification of the number of migrated DPT in the lower chamber after HBEGF knockdown in endothelial cells. (I–K) RT‐qPCR analysis of immune suppression‐related genes (I), epithelial–mesenchymal transition‐related genes (J) and stemness‐associated genes (K) in Hep3B cells after co‐culture with DPTs. (L) Representative images of wound‐healing assays in Hep3B cells after co‐culture with DPTs. (M) Quantification of wound closure in (L). NC, negative control; ns, not significant; RT‐qPCR, reverse transcription quantitative PCR. *p < .05, **p < .01, ***p < .001.

To further examine the relationship between DPT cells and ECs, paired peripheral blood and tumour samples from patients with HCC were analysed. The flow cytometric gating strategy for DPT cell analysis and sorting is shown in Figure 8A. DPT cells were detected in both peripheral blood and tumour tissues, and their proportion among T cells did not differ significantly between the two compartments in three paired samples (Figure 8B). In peripheral blood samples from another four patients with HCC, paired analysis showed that the proportion of PD‐1^+^ cells was significantly higher in DPT cells than in CD8^+^ T cells (Figure 8C,D). HBEGF was then knocked down in AW‐CCH599 ECs using siRNAs, and both RT‐qPCR and western blot confirmed effective silencing. Based on the knockdown efficiency, si‐HBEGF #1 was used in subsequent experiments (Figure 8E,F).

A Transwell migration assay was established to assess whether endothelial‐derived HBEGF influences DPT migration (Figure 8G). Silencing HBEGF in ECs significantly reduced the number of DPT cells recovered from the lower chamber (Figure 8H), supporting a role for endothelial‐derived HBEGF in regulating DPT migration in vitro. In separate non‐contact co‐culture experiments, DPT cells increased the mRNA expression of several immune suppression‐related genes, including CD155, CCL5, CCL22 and CD274, as well as epithelial–mesenchymal transition‐related genes, including CDH2, VIM, SNAIL1 and SLUG, in Hep3B cells (Figure 8I,J). In addition, DPT co‐culture increased the mRNA expression of stemness‐associated genes, including CD44, OCT4 and CD133 (Figure 8K). Consistently, wound‐healing assays showed that co‐culture with DPT cells increased Hep3B cell migration, as reflected by greater wound closure at 24, 48 and 72 h (Figure 8L,M).

## 4 DISCUSSION

The TME plays a pivotal role in determining the prognostic heterogeneity of HCC patients. In this study, we integrated CyTOF data from the Xiangya cohort and the Fudan–Huashan cohort (totalling 51 patients and 58 samples) with single‐cell and bulk RNA sequencing to uncover key immune cell subsets and key molecules associated with differential prognosis in HCC. Through stringent quality control and multi‐modal integration, we delineated HCC‐associated cellular features at single‐cell resolution and identified previously underappreciated hybrid cell populations, including DPT cells as well as CD45^+^EpCAM^+^ and CD45^+^α‐SMA^+^ subsets. These atypical populations, which are often overlooked in conventional analyses, were consistently observed across independent cohorts. By integrating pathway enrichment analysis, cell–cell interaction inference, and spatial transcriptomic profiling, we explored the functional properties and spatial organisation of DPT cells. Importantly, leveraging an independent HCC cohort receiving T+A therapy (first‐line standard systemic therapy for unresectable HCC), we further assessed the potential clinical relevance of DPT cells. To further explore the functional relevance of these observations, additional in vitro assays were performed. Endothelial‐derived HBEGF knockdown reduced DPT migration in a Transwell system, supporting a role for HBEGF in regulating DPT migratory behaviour. In separate co‐culture experiments, DPT cells induced the transcriptional up‐regulation of immunosuppression‐related, EMT‐related and stemness‐associated genes in Hep3B cells and enhanced tumour‐cell migration in wound‐healing assays. These findings provide preliminary functional support for the association between DPT cells and a pro‐TME, but they do not yet establish a complete causal axis in vivo.

CD4^+^CD8^+^ T cells were previously described as abnormal cells which had escaped from faulty thymic selection.^28^ It has also been reported that DPT cells can arise from CD8^+^ T cells under specific conditions, highlighting the plasticity of T cell differentiation.^29^ Recent studies have shown that DPT cells exist in renal cell carcinoma,^30^ lung cancer,^28^ melanoma tissue^28^ and urothelial bladder cancer tumours.^31^ They are considered the result of antigen‐stimulated single positive T‐cell differentiation. The presence of DPT cells in the TME may be partially attributed to their recruitment from peripheral blood, given their relatively higher proportion in circulation compared with liver tissues. This observation suggests a potential trafficking of DPT cells from the periphery into tumour sites, although their local differentiation and phenotypic plasticity within the hepatic TME cannot be excluded. DPT cells express high levels of immune checkpoints^30^ and favour T helper‐2 polarisation in urological cancers.^31^ In murine melanoma, DPT cells have been shown to possess a distinct phenotype characterised by functional impairment, proliferative suppression, and sustained cytotoxicity.^28^ Our results indicate that a higher proportion of DPT cells is associated with poorer prognosis in HCC patients. Mechanistically, DPT cells enhanced the migration of HCC cell lines in vitro and up‐regulated the mRNA expression of genes associated with stemness, EMT and immunosuppression. However, DPT cells exhibit MHC restriction and enhanced cytotoxic potential.^28^ A previous study identified 11 DPT clusters in the leading‐edge regions of HCC tumours, and patients with high levels of PD‐1^+^ DPT cells, which represented a functionally active state, exhibited better prognosis.^11^ Several methodological and conceptual differences may account for these discrepancies. First, the prior study relied on immunohistochemical‐based quantification of limited marker‐defined populations, whereas our study employed high‐dimensional mass cytometry in combination with scRNA‐seq, enabling a more comprehensive and unbiased characterisation of the DPT compartment. Second, whereas previous work focused on PD‐1^+^ DPT cells, we evaluated the overall abundance of intra‐tumoural DPT cells and further resolved their heterogeneity. Notably, we found that DPT cells comprise three distinct states: naïve, activated, and exhausted. The exhaustion phenotype of DPT cells appears to be linked to unfavourable outcomes. Our data refine this view by demonstrating that exhausted DPT cells are preferentially enriched in tumours from short‐survival patients, suggesting that the clinical impact of DPT cells is highly dependent on their functional state. This functional heterogeneity may partly explain the discrepant prognostic associations of DPT cells reported across different studies and patient cohorts. In the T+A‐treated cohort, patients with higher DPT scores exhibited significantly better therapeutic responses, indicating that DPT score may serve as a potential predictive biomarker for treatment efficacy. Enrichment results showed that immunotherapy‐relevant signalling, including PD‐L1‐associated signatures and the PD‐1 checkpoint pathway, was preferentially represented in DPT cells. The functional states of DPT cells are likely shaped by their interactions with surrounding immune and stromal components within the TME. Single‐cell communication analyses stratified by short‐ and long‐term survival revealed that capillary‐associated ECs, monocytes and M2 macrophages exhibited markedly stronger bidirectional interactions with DPT cells than other cellular populations. Accordingly, we further leveraged spatial transcriptomics to focus on the relationships among capillary‐associated ECs, M2 macrophages and DPT cells, given their closer mechanistic relevance to the T+A regimen that simultaneously targets angiogenesis and immune suppression.^32^ Our spatial analyses demonstrated increased DPT infiltration in regions enriched for capillary‐associated ECs and M2 macrophages, suggesting that this niche may represent a critical microenvironmental determinant of unfavourable prognosis and may be amenable to remodelling by T+A therapy. In particular, DPT cells may be involved in shaping a pro‐angiogenic and immunomodulatory TME in HCC. DPT cells may represent a candidate indicator of immunotherapeutic response.

Additionally, we identified HBEGF and CX3CR1 as prognostic risk factors through bulk RNA‐seq analysis. HBEGF expression was significantly and positively correlated with the proportion of DPT cells in both cohorts. In vitro, knockdown of endothelial‐derived HBEGF reduced DPT migration, further supporting its role in DPT recruitment. HBEGF expression has been reported to correlate with microvascular density in HCC, and this molecule is considered a tumour‐promoting factor that may be therapeutically targeted to inhibit angiogenesis.^33^ Single‐cell profiling revealed that HBEGF was predominantly expressed in myeloid and endothelial compartments, with high expression in capillary‐associated ECs, artery ECs, M2 macrophages and monocytes; together with our cell–cell communication and spatial transcriptomic findings and the relative enrichment of EGFR (the receptor for HBEGF) in DPT cells among T‐cell subsets, these data suggest that ECs and M2 macrophages may contribute to DPT accumulation and niche formation, with HBEGF representing a candidate mediator that warrants further validation. By contrast, the role of CX3CR1 remains correlative in the present study and requires direct functional validation. However, CX3CR1 was significantly positively correlated with the proportion of DPT cells only in the Xiangya cohort, whereas this association was not observed in the external cohort, which may be attributable to limited sample size; nevertheless, additional analyses were performed to explore its potential role. At the single‐cell level, CX3CR1 was highly expressed in GZMB^+^ CD8^+^ T cells and CD56^dim^ NK cells, and these two populations were preferentially enriched in the short‐term survival group. Notably, CX3CL1 (the ligand for CX3CR1) showed the highest expression in DPT cells compared with other T‐cell subsets, implying a potential CX3CL1–CX3CR1 communication axis linking DPT cells with GZMB^+^ CD8^+^ T cells and CD56^dim^ NK cells. CX3CR1^34^ promotes recruitment of pro‐angiogenic tumour associated macrophages (TAMs) and enhances EC migration and tube formation.

CD45RA serves as a marker for naive T cells. Long‐lived antigen‐specific memory T cells are characteristically marked by CD161 expression.^35^ CD45RA^+^CD4^+^ Tconv cells could represent naive conventional CD4^+^ T cells characterised by limited effector function. Their enrichment in tumours with poor prognosis may reflect an immune microenvironment dominated by poorly differentiated or functionally inactive T cells, indicating insufficient anti‐tumour immunity. CD161^+^CD45RA^−^CD4^+^ T cells could denote active or memory conventional CD4^+^ T cells. It has been reported that CD45RA^+^CD4^+^ Tconv cells are frequently induced to express Foxp3 in the TME, thereby exerting a pro‐tumourigenic effect.^36^, ^37^ This is consistent with our results. CD161^+^CD45RA^−^CD4^+^ T cells identified in our study exhibited phenotypic features resembling MAIT cells, particularly their high expression of CD161 and memory‐like characteristics. Among T‐cell subsets, MAIT cells are known to display substantially higher CD161 expression than conventional T cells. Accumulating evidence indicates that the functional roles of MAIT cells in cancer are highly heterogeneous and remain controversial.^38^ This apparent inconsistency highlights the context‐dependent nature of MAIT cell effector functions, which may explain their dichotomous properties across different TMEs.^38^

We identified two previously uncharacterised cell clusters, Cluster 14 and Cluster 34. The two clusters were significantly associated with patient outcomes, as validated by scRNA‐seq and RNA‐seq analysis. They may be new cell clusters induced by antigens or cell clusters with overlapping phenotypes. In tumours, EpCAM is expressed almost exclusively on epithelial‐derived tumour cells, while CD45 is expressed exclusively on immune cells. Cluster 14, characterised by co‐expression of CD45 and α‐SMA, exhibited a gene expression pattern resembling that of apCAFs and may promote tumourigenesis via immune checkpoint‐related mechanisms. Cluster 34, defined by co‐expression of EpCAM and CD45, displayed a gene expression profile similar to epithelial C8 cells and may exert pro‐tumourigenic effects through the PPAR signalling pathway. The possible explanation is that some immune cells with phagocytic function express epithelial markers on the cell surface by phagocytosis of tumour‐secreted vesicles. Another explanation is that tumour cells mimic immune cells, which may be associated with cell fusion.^39^, ^40^ And this fusion cell may be associated with tumour metastasis, immune escape and stem cell enhancement.^39^, ^41^, ^42^, ^43^, ^44^ How this novel phenotypic cell population arises remains unknown.

Even with the multi‐omics framework applied here, the present study remains subject to several limitations. First, the CyTOF‐based discovery cohort comprised a relatively modest number of patients, which may introduce selection bias, limit statistical power and affect the stability of effect‐size estimation. This constraint is inherent to high‐dimensional immune profiling studies using fresh tumour tissues, where tissue availability and stringent quality control requirements restrict sample size. To mitigate these limitations, we adopted a multi‐level validation strategy, including an independent CyTOF cohort, bulk RNA sequencing, single‐cell RNA sequencing, multiplex immunohistochemistry, and survival analyses in the TCGA‐LIHC cohort. The consistency of key prognostic associations across independent cohorts and orthogonal platforms supports the robustness of the identified immune features. Second, the additional functional assays were mainly in vitro and based on a limited number of patient‐derived DPT samples, which may limit generalisability. Moreover, HBEGF was only partially validated, whereas CX3CR1 remained correlative. Third, several important clinicopathological variables, including liver function status, AFP levels, tumour burden and vascular invasion, were unavailable in some public cohorts, limiting comprehensive multivariate adjustment for potential confounding factors. While we have now performed additional functional experiments to support the biological relevance of the key findings, these assays remain limited in scope and do not fully establish causality or delineate the complete underlying mechanisms. In addition, the immunotherapy analysis was based on biomarker‐evaluable pretreatment transcriptomic subsets from the GO30140 and IMbrave150 trials, and residual inter‐trial heterogeneity may still exist despite batch correction. Therefore, the results should still be interpreted primarily as robust association‐based evidence with partial functional support, rather than fully mechanistic conclusions. Future studies using larger, prospectively collected cohorts and more comprehensive mechanistic models will be required to further validate these observations and clarify their biological implications.

In conclusion, we delineated prognosis‐associated immune cell subsets and molecular features within the HCC TME through integrated multi‐omics analyses. We found that intra‐tumoural DPT cells were consistently associated with unfavourable prognosis across multiple cohorts. Functional assays further provided preliminary support for a potential link between endothelial HBEGF, DPT‐cell migration and tumour‐promoting effects in vitro. Collectively, our findings suggest that DPT cells may serve as a candidate prognostic biomarker and that the HBEGF‐related DPT niche warrants further investigation in HCC, particularly in the context of anti‐angiogenic and immunotherapeutic strategies.

## AUTHOR CONTRIBUTIONS

G. C. and E. H. conceived and designed the study. Q. P., X. Z., Y. Z., H. L. and X. W. performed the experiments and collected the data. G. C., E. H. and Y. Z. carried out the bioinformatics and statistical analyses. G. C. and E. H. drafted the manuscript. Y. C., S. Z. and H. S. provided critical revision of the manuscript. Y. C., S. Z. and H. S. acquired funding. S. Z. and J. M. supervised the project. All authors read and approved the final manuscript.

## FUNDING

This study was supported by grants from the National Natural Science Foundation of China (grant number 82373275 and 82173342), the Natural Science Foundation of Hunan Province (grant number 2025JJ60506) and Wu Jieping Medical Foundation (grant number 320.6750).

## ETHICS STATEMENT

This study was approved by the Ethics Committee of Xiangya Hospital, Central South University (Approval No. 202103218). Written informed consent was obtained from all participants prior to sample collection. All animal experiments were performed in accordance with the Guidelines for the Welfare and Ethical Review of Laboratory Animals (GB/T 35892‐2018; effective 1 September 2018) and internationally accepted standards for animal research. The study protocol was reviewed and approved by the Experimental Animal Welfare and Ethics Review Committee of Xiangya Hospital, Central South University.

## CONFLICT OF INTEREST STATEMENT

The authors declare no conflicts of interest.

## Supporting information



Supporting Information

Supporting Information

## Data Availability

All datasets presented in this study are included in the article/Supplementary Material. Further inquiries can be directed to the corresponding author.
