# Supplementary material for: Multi‐omics insights for deciphering prognosis‐related T cell subsets in hepatocellular carcinoma
Source: Clin Transl Med. 2026 Jun 1;16(6):e70708. doi: 10.1002/ctm2.70708 (PMC13239885; doi:10.1002/ctm2.70708)
Supplement: Supplementary file 1 — Supporting Information [file CTM2-16-e70708-s002.docx]

**Cell culture, siRNA transfection, and Transwell-based migration/co-culture assays**

AW-CCH599 endothelial cells were maintained in AW-MC013 medium. Hep3B cells were maintained in DMEM supplemented with 10% fetal bovine serum (FBS) and 1% penicillin-streptomycin. DPT cells sorted from patient samples were cultured in RPMI-1640 medium supplemented with 10% FBS, 1% penicillin-streptomycin, 50 μM β-mercaptoethanol, and recombinant human IL-2, and were stimulated with anti-CD3/CD28 beads for expansion. All cells were cultured at 37°C in a humidified incubator with 5% CO2. All the cell lines were purchased from the Institutes of Biomedical Sciences.

For siRNA transfection, AW-CCH599 cells were seeded one day before transfection at an initial density of 1×10^5 cells per well and transfected at approximately 60% confluence. HBEGF-targeting siRNAs and negative control siRNA were designed and synthesized by Sangon Biotech (Shanghai, China). The sense sequences of HBEGF-targeting siRNAs were as follows: si-HBEGF #1, 5′-GGAGAAUGCAAAUAUGUGAdTdT-3′; si-HBEGF #2, 5′-GAAAGUCCGUGACUUGCAAdTdT-3′; si-HBEGF #3, 5′-GAGGAGGUUAUGAUGUGGAdTdT-3′. Transfection was performed using Lipofectamine™ 3000 reagent (Invitrogen, Thermo Fisher Scientific, Waltham, MA, USA) according to the manufacturer’s instructions. Briefly, siRNA and Lipofectamine™ 3000 reagent were diluted separately in Opti-MEM™ Reduced Serum Medium, incubated for 5 min, gently mixed, and allowed to stand at room temperature for 15 min before being added to the cells. After 6 h, the medium was replaced with fresh complete medium, and the cells were cultured for subsequent experiments.

To evaluate endothelial cell-mediated DPT migration, Transwell inserts containing 5.0 μm pore polycarbonate membranes (24-well format, 6.5 mm diameter) were used. AW-CCH599 cells were seeded in the lower chamber at 1×10^5 cells per well. After the cells reached approximately 60% confluence, siRNA transfection and medium replacement were performed as described above. DPT cells (1×10^5) were then added to the upper chamber. After 6 h, the lower-chamber supernatant was collected, and adherent cells in the lower chamber were harvested by digestion. The two fractions were pooled and analyzed by flow cytometry. Because AW-CCH599 cells are CD45-negative, CD45^+^ cells recovered from the lower chamber were regarded as migrated DPT cells and used for quantitative analysis.

For non-contact co-culture, Transwell inserts containing 0.4 μm pore PET membranes (24-well format, 6.5 mm diameter) were used. Hep3B cells were seeded in the lower chamber at 1×10^5 cells per well and cultured to approximately 60% confluence. DPT cells (1×10^5) were then added to the upper chamber, and the two cell populations were co-cultured for 48 h. Hep3B cells in the lower chamber were subsequently collected for downstream analyses.

For the wound-healing assay, Hep3B cells were seeded in the lower chamber of 24-well plates at 1×10^5 cells per well and co-cultured with DPT cells in the upper chamber using 0.4 μm pore Transwell inserts. After 48 h of co-culture, the upper inserts were removed and a linear scratch was made in the Hep3B monolayer. The cells were then maintained in low-serum medium, and wound closure was monitored up to 72 h. Images were acquired at the indicated time points, and the percentage of wound closure was calculated for statistical analysis.

**RT-qPCR**

Total RNA was isolated with the SteadyPure Quick RNA Extraction Kit (AG21023, Accurate Biology, Changsha, China) following the manufacturer’s protocol. cDNA was generated using the Evo M-MLV RT Mix Kit with gDNA Clean for qPCR Ver.2 (AG11728, Accurate Biology, Changsha, China) in a 20 μl reaction volume. Quantitative PCR was performed with an Accurate Biology SYBR Green qPCR premix in a total volume of 20 μl. The amplification program consisted of 95°C for 30s, followed by 40 cycles of 95°C for 5s and 60°C for 30s. A melting curve was subsequently generated at 95°C for 15s, 60°C for 1min, and 95°C for 1s. Primer sequences are provided in Supplementary Table 13. Relative mRNA expression levels were normalized to β-tubulin and calculated using the 2^−ΔΔCt^ method.

**Western blot**

Cells were washed once with pre-chilled PBS and lysed on ice in RIPA buffer supplemented with protease inhibitors for 30min. During lysis, samples were mixed every 5–10min to ensure complete disruption. After centrifugation at 12,000rpm for 15min at 4°C, the supernatants were collected as total protein extracts. Protein concentration was measured using a BCA protein assay kit, and the lysates were adjusted to the same concentration. Equal amounts of protein were mixed with 5×loading buffer, heated at 95°C for 5min, and subjected to SDS-PAGE, followed by transfer onto PVDF membranes. After activation in methanol and equilibration in transfer buffer, the membranes were blocked with 5% BSA for 1 h at room temperature and then incubated overnight at 4°C with anti-HBEGF antibody (AWA11948, Abiowell). After washing three times with TBST, the membranes were incubated with HRP-conjugated goat anti-rabbit IgG (SA00001-2, Proteintech) for 1h at room temperature. For loading control detection, membranes were incubated with HRP-conjugated β-tubulin antibody (HRP-66240, Proteintech). After washing with TBST, protein bands were visualized using enhanced chemiluminescence and captured with a gel imaging system.


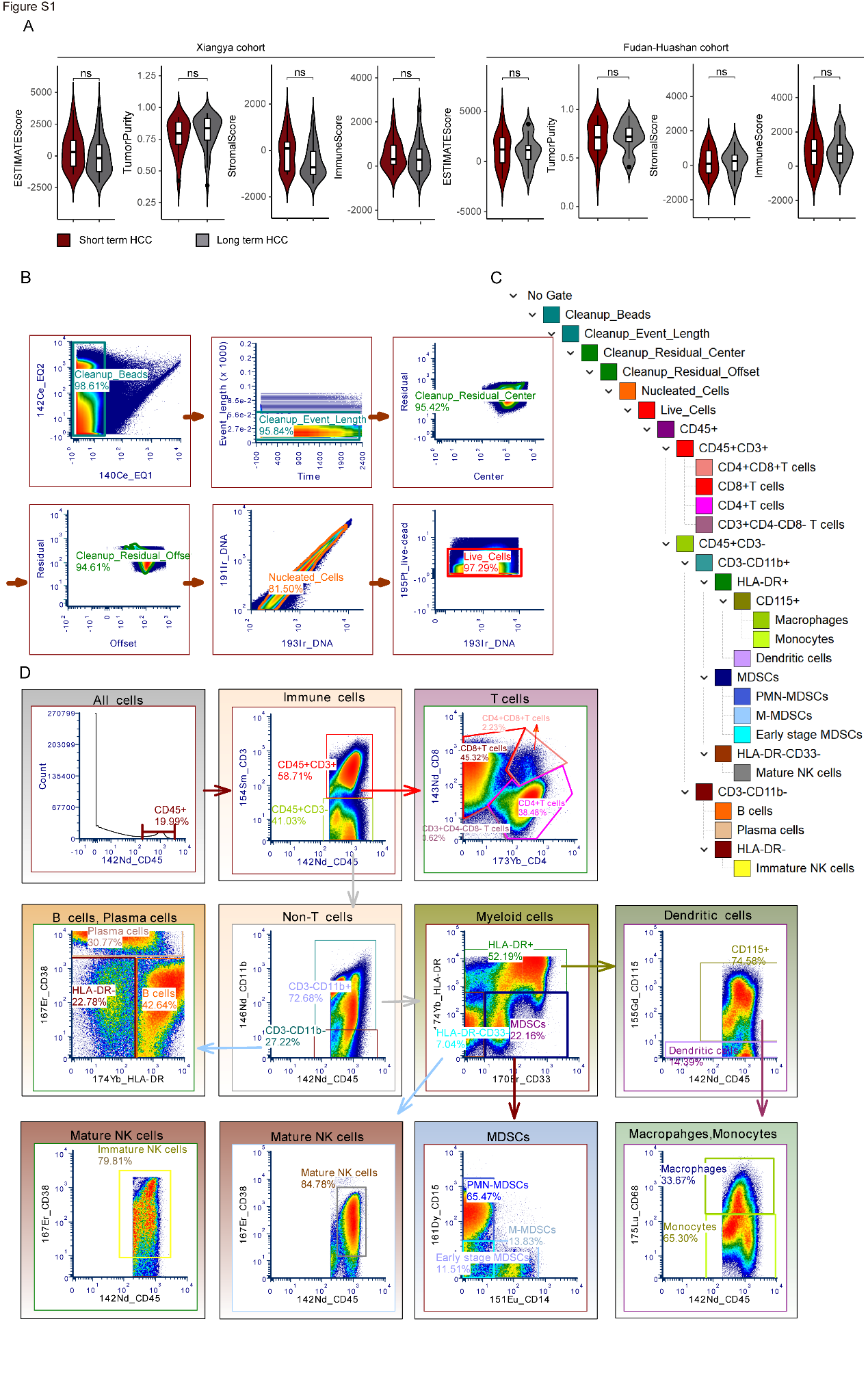


**Fig. S1. Gating strategy for identifying major immune cell groups in the Xiangya cohort.**

A. ESTIMATE score, tumor purity, stromal score, and immune score in patients with short- and long-term survivors in the Xiangya cohort and the Fudan-Huashan cohort.

B. Gating workflow for mass cytometry data quality control in the Xiangya cohort.

C. Gate hierarchy for mass cytometry analysis.

D. Gating strategy for major immune cell populations in the Xiangya cohort.


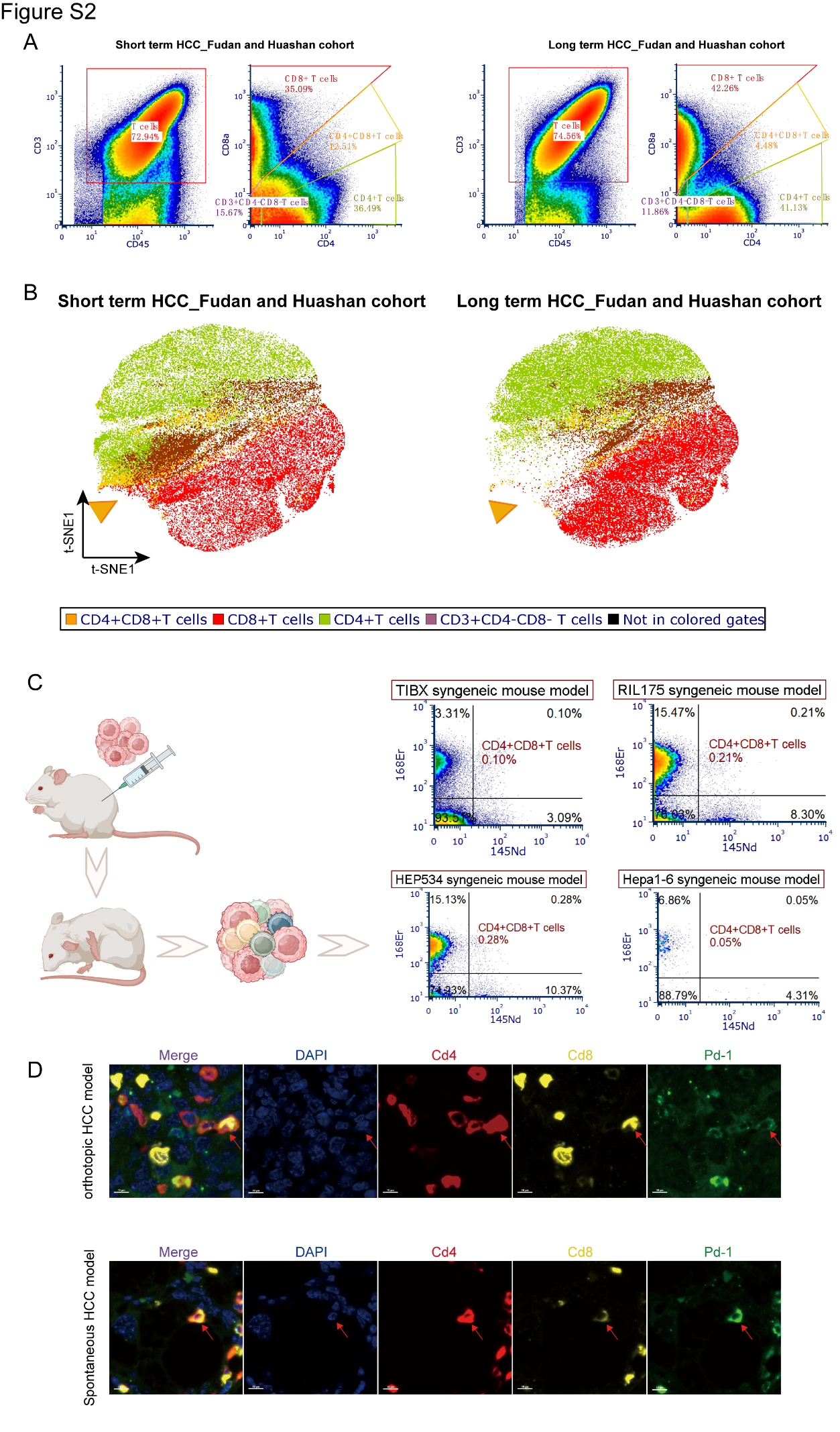


**Fig. S2. Tumor-infiltrating DPT cells are validated in an independent clinical cohort and syngeneic mouse models.**

A. Density plot of mass cytometry data showing tumor-infiltrating DPT cells in patients with short- and long-term survivors in the Fudan-Huashan cohort.

B. Dimensionality reduction showing DPT cells in tumors from patients with short- and long-term survivors in the Fudan-Huashan cohort.

C. Density plots showing tumor-infiltrating DPT cells in TIBX, RIL175, HEP534, and Hepa1-6 syngeneic mouse models.

D. Representative mIHC images highlighting PD-1^+^ DPT cells in orthotopic and spontaneous hepatocellular carcinoma mouse models.


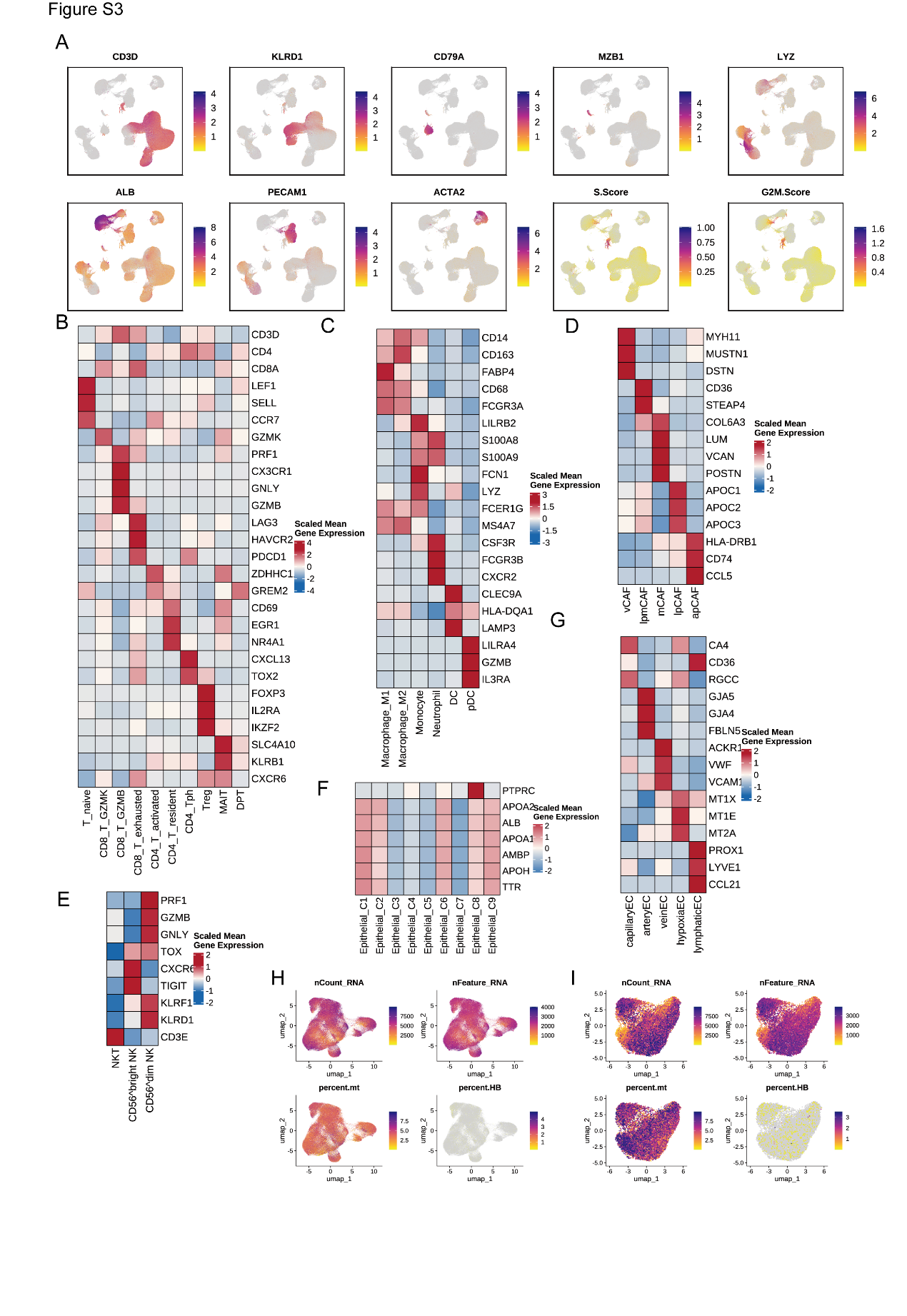


**Fig. S3. Marker validation and quality-control metrics for scRNA-seq cell-type annotation.**

A. Feature plots showing representative lineage marker expression across the UMAP embedding, together with cell-cycle score.

B-G. Heatmaps of scaled mean expression of canonical marker genes used to define cell subsets, including T-cell subsets (B), myeloid subsets (C), CAF subsets (D), NK-cell subsets (E), epithelial clusters (F), and endothelial subsets (G) in the PRJCA007744 dataset.

H-I. UMAP projections of quality-control metrics for T cells (H) and DPT cells (I), including nCount_RNA, nFeature_RNA, percent.mt, and percent.HB.


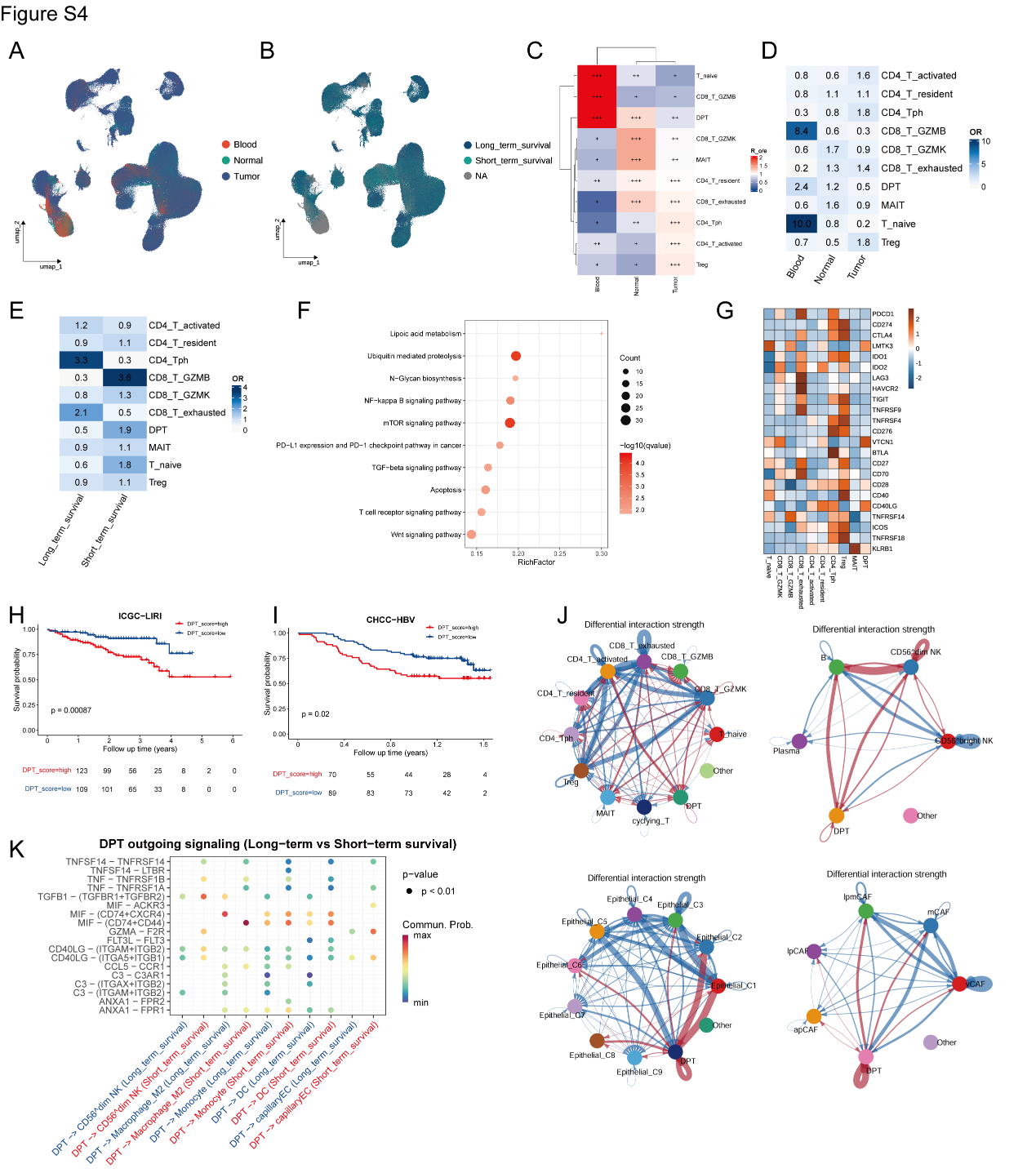


**Fig. S4. Tissue- and prognosis-associated T-cell landscape highlights DPT heterogeneity, functional programs, survival relevance, and intercellular communication.**

A. UMAP visualization of single cells grouped by tumor, adjacent non-tumor tissue, and blood samples from the PRJCA007744 dataset.

B. UMAP visualization of single cells grouped by short- and long-term survivors.

C. ROE heatmap of tissue preference for T-cell subsets across blood, adjacent non-tumor tissue, and tumor.

D. OR heatmap of tissue preference for T-cell subsets across blood, adjacent non-tumor tissue, and tumor.

E. OR heatmap of tissue preference for T-cell subsets in patients with short- and long-term survivors.

F, KEGG enrichment bubble plot of DPT differentially expressed genes.

G. Heatmap of immune checkpoint expression across T-cell subsets.

H-I. Kaplan-Meier survival curves stratified by DPT signature ssGSEA score in the ICGC-LIRI (H) and CHCC-HBV (I) cohorts.

J. Compartment-level CellChat differential interaction networks (short- and long-term survivors) from the PRJCA007744 dataset. Edge width indicates differential interaction strength. Red indicates stronger interactions in short-term survivors, and blue indicates stronger interactions in long-term survivors.

K. CellChat bubble plots of DPT outgoing signaling.

ROE, relative odds enrichment. OR, odds ratio.


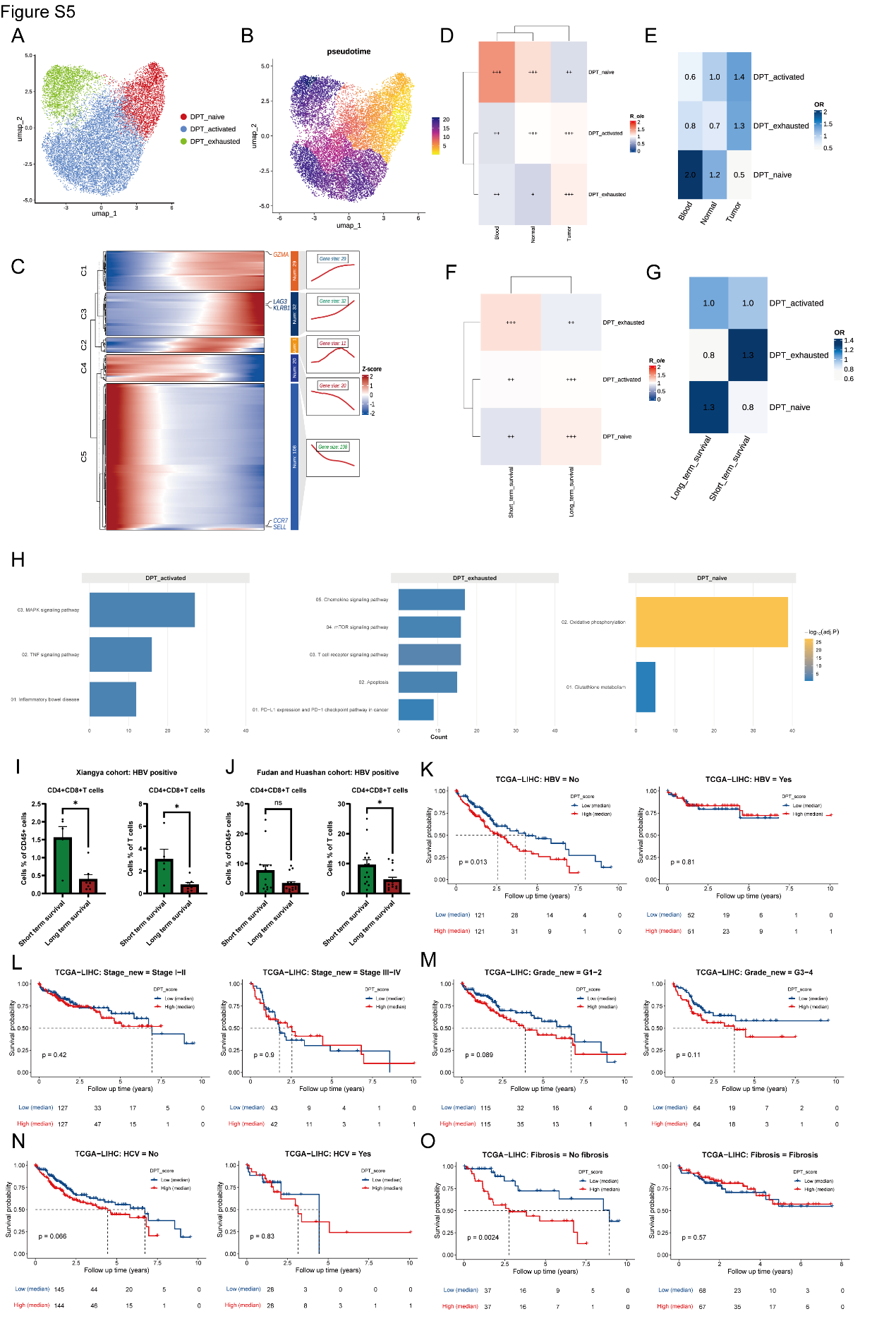


**Fig. S5. Trajectory-defined DPT states associate with tissue enrichment and survival across clinical subgroups in HCC.**

A. UMAP visualization of DPT cells showing three DPT states from the PRJCA007744 dataset.

B. UMAP visualization of DPT cells colored by pseudotime.

C. Pseudotime heatmap of gene modules (C1-C5), with cells ordered left-to-right along the trajectory.

D. ROE heatmap of tissue preference for DPT states across blood, adjacent non-tumor tissue, and tumor.

E. OR heatmap of tissue preference for DPT states across blood, adjacent non-tumor tissue, and tumor.

F, ROE heatmap showing tissue preference of DPT states in short-term versus long-term survival groups.

G. OR heatmap of tissue preference for DPT states in patients with short- and long-term survivors.

H. KEGG enrichment analysis of differentially expressed genes across distinct DPT states.

I-J. Comparison of DPT cell frequencies between short- and long-term survivors with HBV infection in the Xiangya (I) and Fudan-Huashan (J) cohorts.

K-O. Kaplan-Meier survival analysis stratified by DPT score in the TCGA-LIHC cohort: stage I-II, stage III-IV (L), grade 1-2, grade 3-4 (M), without HBV infection , with HBV infection (K), with HCV infection, without HCV infection (N), with fibrosis, and without fibrosis (O).

ROE, relative odds enrichment. OR, odds ratio.


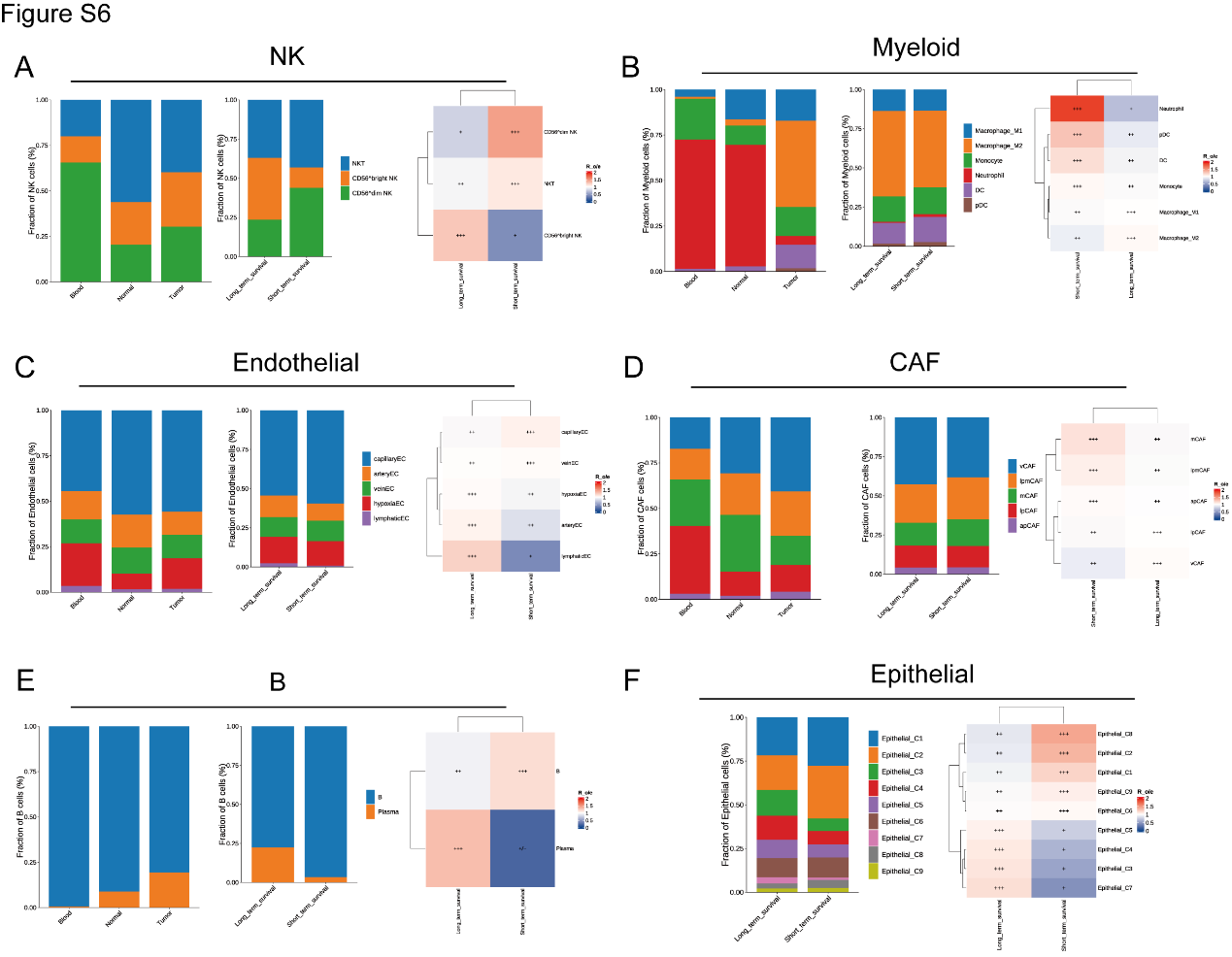


**Fig. S6. Cell-type-specific compositional shifts and survival-associated tissue preference of non-T-cell subsets.**

For each major compartment (A, NK; B, myeloid; C, endothelial; D, CAF; E, B/plasma; F, epithelial), stacked bar plots show subset fractions across blood/adjacent non-tumor/tumor (left) and across short- and long-term survivors (middle) from the PRJCA007744 dataset. ROE heatmaps comparing short- and long-term survivors are shown on the right.


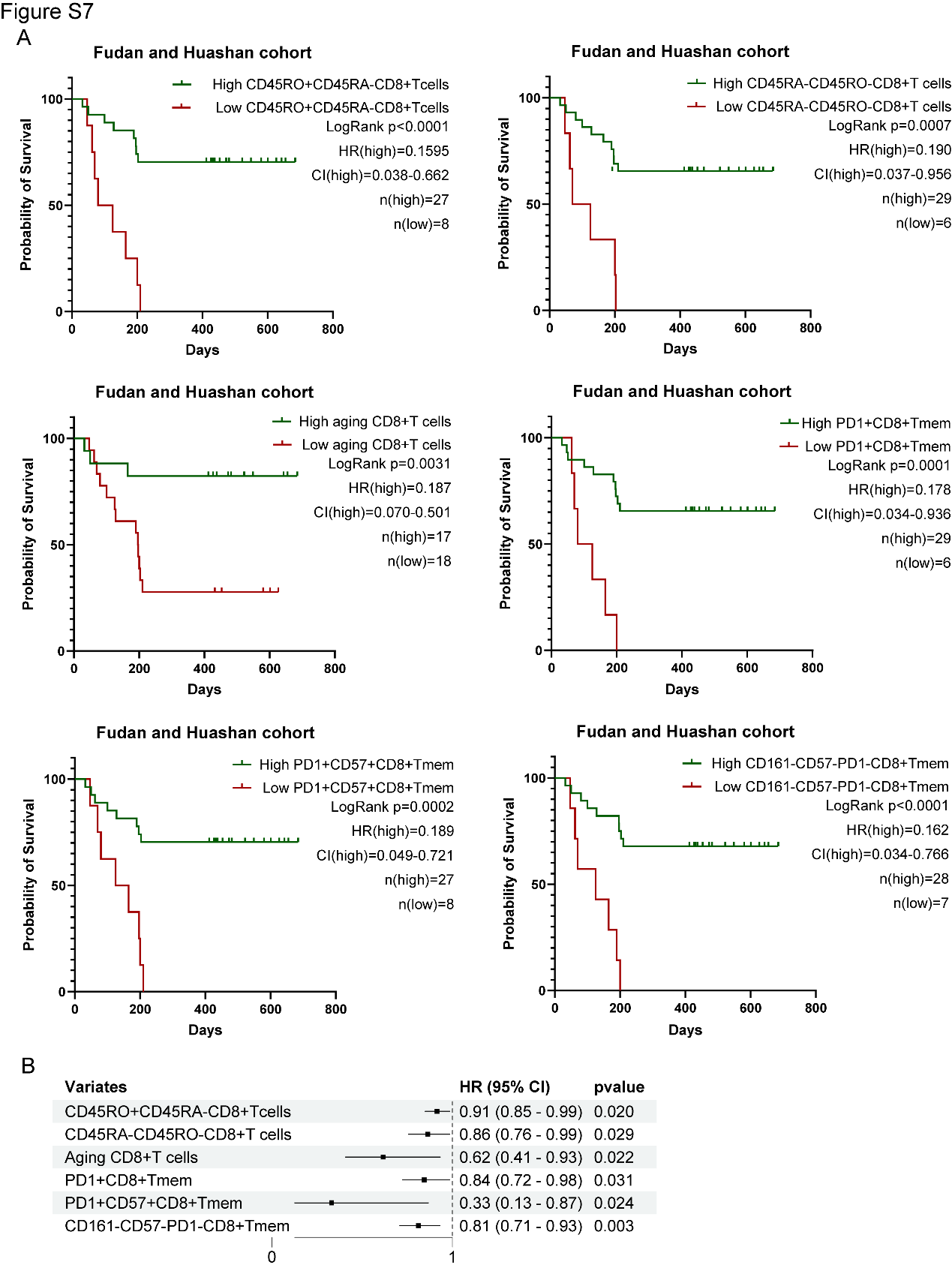


**Fig. S7. Tumor-infiltrating CD8^+^ T-cell subgroups are protective factors for patients with HCC.**

A. Kaplan-Meier survival analysis with log-rank test in the Fudan-Huashan cohort evaluating the prognostic significance of intratumoral CD8^+^ T-cell subsets.

B. Univariate Cox proportional hazards regression in the Fudan-Huashan cohort evaluating the prognostic impact of tumor-infiltrating CD8^+^ T-cell subsets.

HR, hazard ratio. CI, confidence interval.


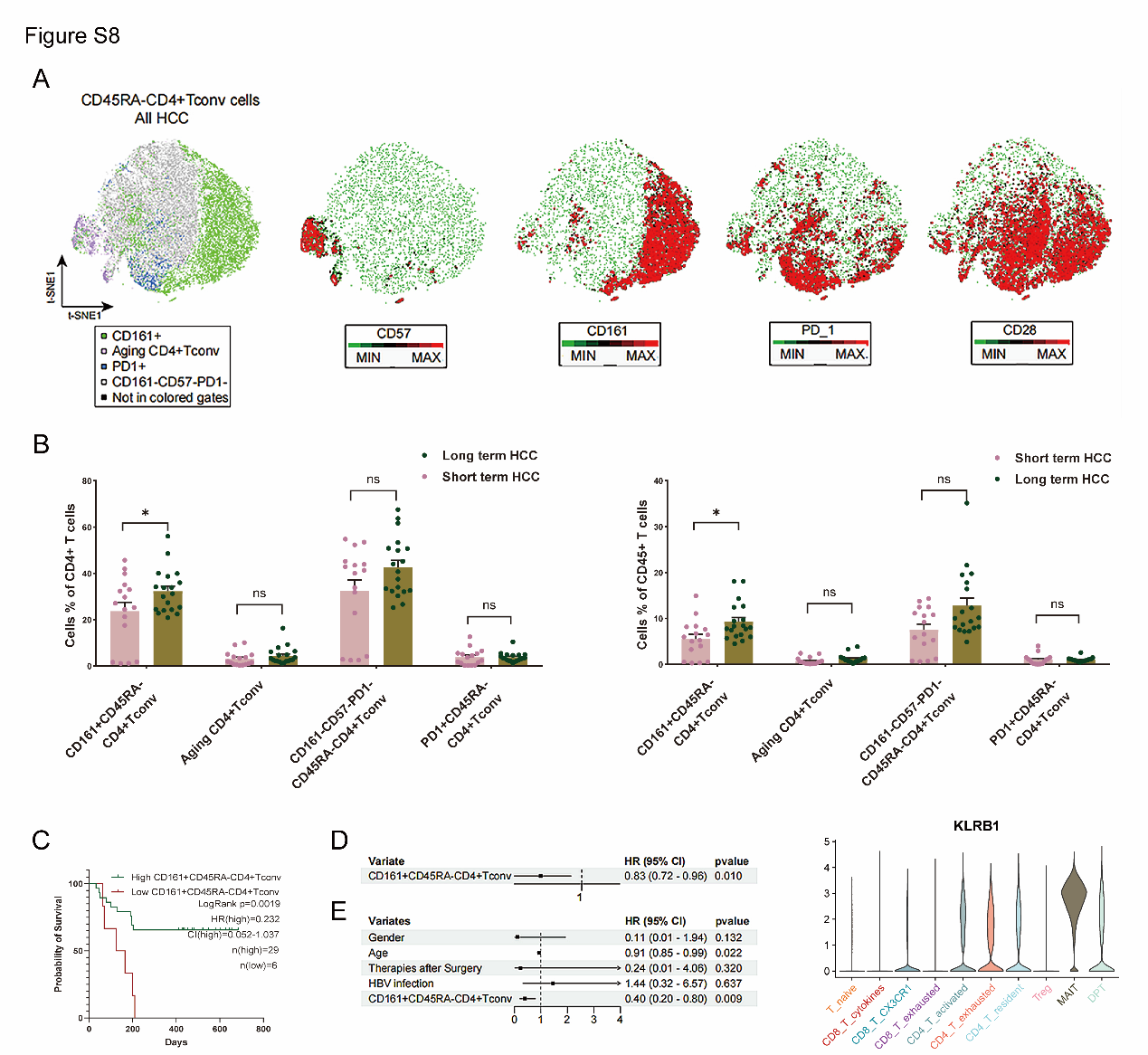


**Fig. S8. Tumor-infiltrating CD161^+^CD45RA^-^ CD4^+^ conventional T cells are decreased in short-term survivors and are associated with favorable prognosis in HCC.**

A. t-SNE projections of tumor-infiltrating CD45RA^-^ CD4^+^ conventional T cells (Tconv) from all survival groups, showing cell composition and marker distribution (CD57, CD161, PD-1, and CD28) from the Fudan-Huashan cohort.

B. Relative frequencies of tumor-infiltrating CD45RA^-^ CD4^+^ Tconv subsets between patients with short- and long-term survivors. Percentages within total CD4^+^ T cells and within CD45^+^ cells are shown.

C. Kaplan-Meier survival analysis with log-rank test in the Fudan-Huashan cohort evaluating the prognostic significance of CD161^+^CD45RA^-^ CD4^+^ Tconv cells.

D. Univariate Cox proportional hazards regression in the Fudan-Huashan cohort evaluating the prognostic significance of CD161^+^CD45RA^-^ CD4^+^ Tconv cells.

E. Multivariate Cox proportional hazards regression in the Fudan-Huashan cohort evaluating the prognostic significance of CD161^+^CD45RA^-^ CD4^+^ Tconv cells.

F. Violin plot showing KLRB1 expression across T-cell subsets.

ns, not significant. *P < 0.05. HR, hazard ratio. CI, confidence interval.


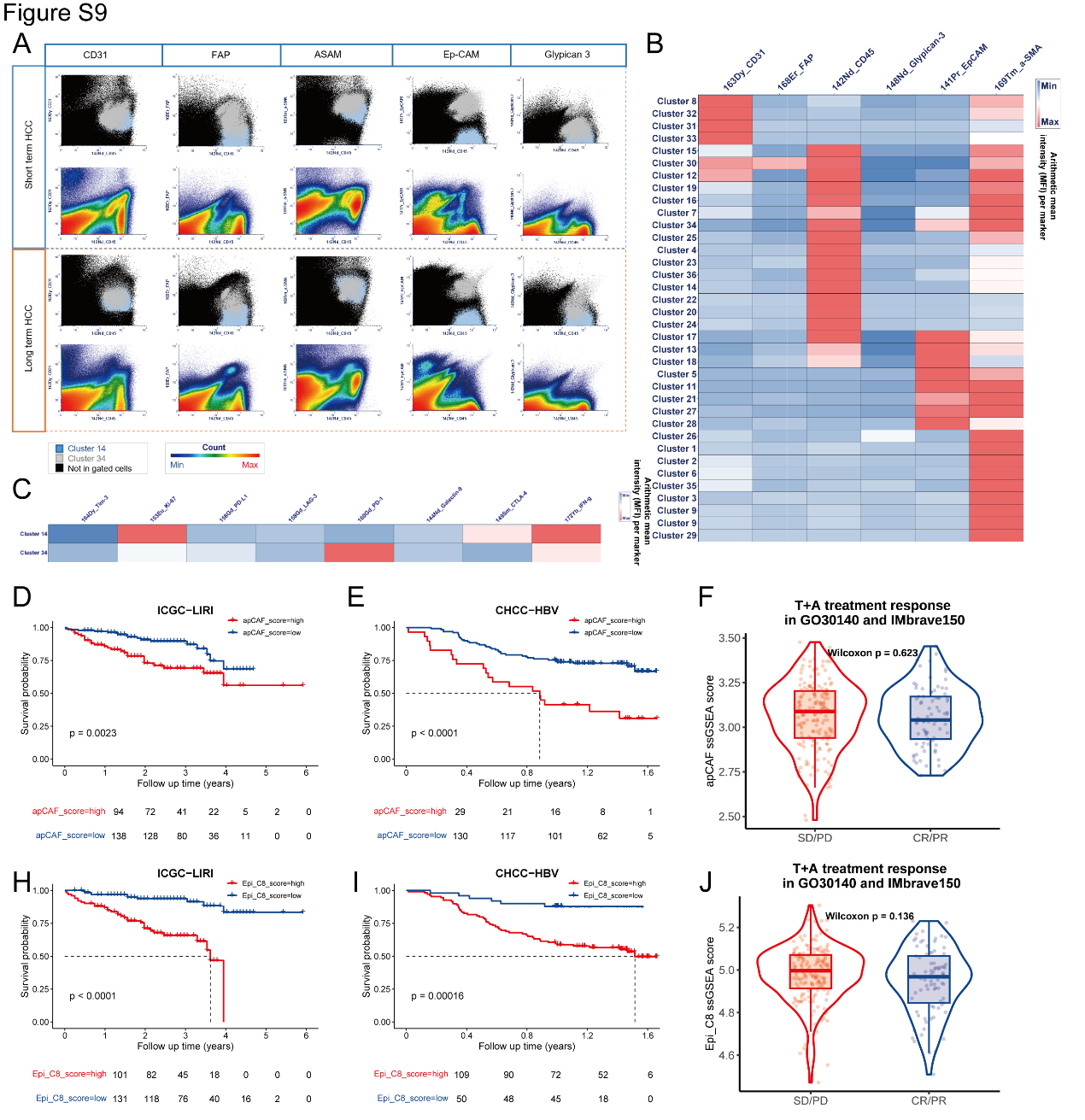


**Fig. S9. Identification and clinical relevance of apCAF and Epithelial_C8 signatures.**

A. Color dot plots and density plots showing expression of CD45, CD31, FAP, alpha-SMA, EpCAM, and Glypican3 in clusters 14 and 34 from the Xiangya cohort.

B. Parameter heatmap of all clusters.

C. Parameter heatmap showing checkpoint expression in clusters 14 and 34.

D-E. Kaplan-Meier survival analysis stratified by apCAF signature ssGSEA score in the ICGC-LIRI (D) and CHCC-HBV (E) cohorts.

F. apCAF signature ssGSEA score versus T+A response in GO30140 and IMbrave150 (SD/PD vs CR/PR).

H-I. Kaplan-Meier survival analysis stratified by Epithelial_C8 signature ssGSEA score in the ICGC-LIRI (H) and CHCC-HBV (I) cohorts.

J. Epithelial_C8 signature ssGSEA score versus T+A response in GO30140 and IMbrave150 (SD/PD vs CR/PR).

T+A, atezolizumab plus bevacizumab.
